# A bacterial gene-drive system efficiently edits and inactivates a high copy number antibiotic resistance locus

**DOI:** 10.1038/s41467-019-13649-6

**Published:** 2019-12-16

**Authors:** J. Andrés Valderrama, Surashree S. Kulkarni, Victor Nizet, Ethan Bier

**Affiliations:** 1 Tata Institute for Genetics and Society, University of California, San Diego, 9500 Gilman Drive, La Jolla, CA 92093-0349 USA; 2 Collaborative to Halt Antibiotic-Resistant Microbes, Department of Pediatrics, University of California, San Diego, 9500 Gilman Drive, La Jolla, CA 92093-0760 USA; 3 Section of Cell and Developmental Biology, University of California, San Diego, 9500 Gilman Drive, La Jolla, CA 92093-0349 USA; 4 Skaggs School of Pharmacy & Pharmaceutical Sciences, University of California, San Diego, 9500 Gilman Drive, La Jolla, CA 92093-0760 USA

**Keywords:** Antimicrobial resistance, Bacterial genetics, Bacterial techniques and applications, CRISPR-Cas systems, Microbial genetics

## Abstract

Gene-drive systems in diploid organisms bias the inheritance of one allele over another. CRISPR-based gene-drive expresses a guide RNA (gRNA) into the genome at the site where the gRNA directs Cas9-mediated cleavage. In the presence of Cas9, the gRNA cassette and any linked cargo sequences are copied via homology-directed repair (HDR) onto the homologous chromosome. Here, we develop an analogous CRISPR-based gene-drive system for the bacterium *Escherichia coli* that efficiently copies a gRNA cassette and adjacent cargo flanked with sequences homologous to the targeted gRNA/Cas9 cleavage site. This “pro-active” genetic system (Pro-AG) functionally inactivates an antibiotic resistance marker on a high copy number plasmid with ~ 100-fold greater efficiency than control CRISPR-based methods, suggesting an amplifying positive feedback loop due to increasing gRNA dosage. Pro-AG can likewise effectively edit large plasmids or single-copy genomic targets or introduce functional genes, foreshadowing potential applications to biotechnology or biomedicine.

## Abstract (web summary)

Genedrives bias the inheritance of alleles in diploid organisms. Here, the authors develop a gene-drive analogous system for bacteria, selectively editing and clearing plasmids.

## Introduction

Synthetic gene-drive elements based on bacterial-derived CRISPR components have been developed in diploid organisms including yeast^1^, insects^2–4^, and mammals^5^. The salient feature of these “active genetic” systems is that a guide RNA (gRNA) is directly flanked by sequences homologous to the genomic site it targets for Cas9-mediated cleavage. When double-stranded DNA breaks are induced in the germline by the gRNA/Cas9 complex, the gRNA together with any linked cargo sequences are then copied into the break via the homology-directed repair (HDR) pathway^6^. Such gene conversion events can greatly bias transmission of the gRNA cassette so that it is inherited by nearly all progeny.

Echoing their functional CRISPR origins, synthetic bipartite gRNA/Cas9 systems have been developed in bacteria^7–9^, including targeting of plasmid-encoded antibiotic resistance determinants^10^. Indeed, as plasmid-borne antibiotic resistance genes and virulence determinants are important in the pathogenesis of many human bacterial infections, inactivation or replacement of such multi-copy targets could greatly impact the success or failure of treatment interventions^11,12^. We contemplated whether modifying a standard bacterial CRISPR system by developing an active self-copying mechanism could enhance efforts to scrub antibiotic resistance in prokaryotes (“pro-active genetics” or Pro-AG) and thereby address the challenge of high gene dosage on multi-copy plasmids.

Here, we develop a Pro-AG gene-drive system for the bacterium *Escherichia coli* that efficiently copies a functional gRNA cassette flanked with sequences homologous to the targeted gRNA/Cas9 cleavage site. Pro-AG inactivates an antibiotic resistance marker on a high copy number plasmid with ~ 100-fold greater efficiency than control CRISPR-based methods, indicating a self-amplifying positive feedback loop linked to increasing gRNA dosage. Likewise, the system can efficiently edit a large plasmid, target genes on the bacterial chromosome, or be adapted to introduce functional gene cargos alongside the gRNA cassette. Pro-AG expands the available toolkit for engineering or manipulating bacteria in future biotechnology and biomedicine applications.

## Results

### Establishment of the pro-active genetics (Pro-AG) system

**Fig. 1:**
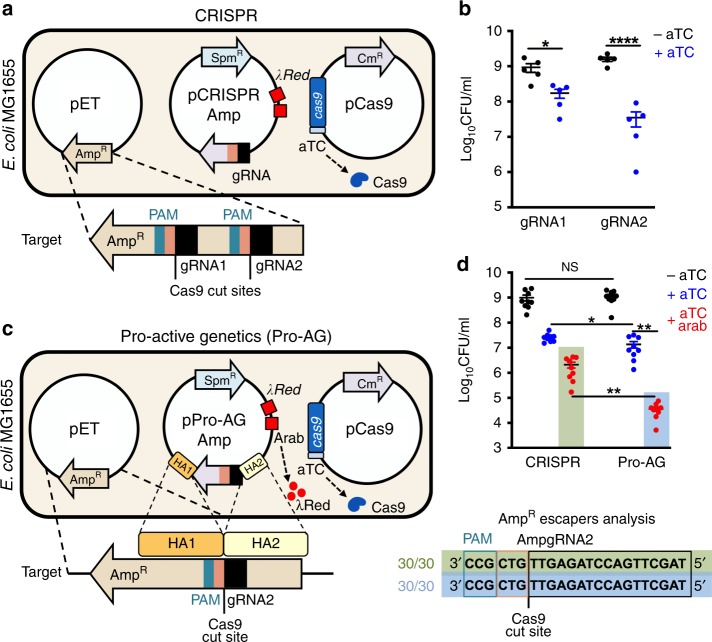
Pro-active genetics (Pro-AG) is ~ 100× more efficient than CRISPR-control for targeting antibiotic resistance conferred by a high copy plasmid. **a** and **c** Schematic of CRISPR-control- **a** and Pro-AG- **c** mediated editing of beta-lactamase gene (*bla*) encoded on a high copy plasmid (pET) conferring resistance to ampicillin (Amp^R^, tan arrow) in *E. coli* MG1655. gRNAs were initially expressed from a low copy plasmid (pCRISPR Amp) maintained under spectinomycin (Spm) selection and using the constitutive *tet* promoter to express the gRNA. A second low copy plasmid, pCas9, maintained under chloramphenicol (Cm^R^) selection, encodes Cas9 under control of an anhydrotetracycline (aTc) inducible promoter. **a** CRISPR-control configuration: two gRNAs (gRNA1 and gRNA2 carried on the pCRISPR Amp plasmid) were tested for Cas9-mediated targeting at two different locations in the *bla* gene. The Cas9/gRNA1 and Cas9/gRNA2-induced cleavage sites, and the protospacer adjacent motif (PAM) are indicated as well as a gene cassette carrying the λRed enzymes. **b** Recovery of Amp^R^ colony-forming units (Log_10_CFU/ml) following CRISPR-mediated targeting of the *bla* gene with gRNAs 1 or 2 in the absence (− aTc, black dots) or presence (+ aTc, blue dots) of aTc-induced Cas9 expression. **c** Pro-AG configuration: the gRNA2 expression cassette flanked by *bla* (Amp^*R*^) homology arms (HA1 and HA2) that directly abut the gRNA2 cleavage site was incorporated into pCRISPR Amp. The Cas9/gRNA2-induced cleavage site, the protospacer adjacent motif (PAM), and the HA1 (dark yellow box), and HA2 (light yellow box) homology arms are indicated. Also carried on plasmids pCRISPR Amp and pPro-AGAmp are the recombinogenic λRed enzymes (λRed), which can be induced with l-arabinose (arab). **d** Recovery of CFU on Amp plates following CRISPR-control versus Pro-AG-mediated targeting of the *bla* gene. In this and subsequent panels, abbreviations for Cas9 induction are as in panel **b**; induction of λRed enzymes: (+ aTc + arab: red dots); CRISPR (green shaded box) and Pro-AG (blue shaded box) treatments are highlighted. Plasmids sequenced from Amp^R^ colonies (CRISPR, green box and Pro-AG, blue box) displayed unaltered gRNA target sites in all clones analyzed (30/30). Data in **b** and **d** are plotted as the mean ± SEM, representing three independent experiments performed in triplicate and analyzed by Student’s *t* test. N.S. = not significant (*P* > 0.05) *P < 0.05; **P < 0.01; ****P < 0.0001. Source Data are available in the Source Data file.

In our first set of experiments, we employed a set of three mutually compatible plasmids to assess Cas9-mediated cleavage/inactivation of the beta-lactamase (*bla*) target gene, which confers ampicillin resistance (Amp^R^) in *Escherichia coli*. The three plasmids were: (1) high copy number pET bearing the *bla* target gene and conferring Amp^R^; (2) low copy pCRISPR Amp carrying one of two different gRNAs targeting *bla* sequences under constitutive transcriptional control and maintained under spectinomycin selection (Spm^R^); and (3) low copy pCas9-expressing Cas9 under control of a *tet* promoter, inducible with anhydrotetracycline (aTc) and propagated under chloramphenicol selection (Cm^R^) (Fig. 1a; Supplementary Table 1).

Initial transformation of *E. coli* with the compatible three-plasmid system (scheme in Supplementary Fig. 1a) did not lead to significant differences in colony-forming unit (CFU) recovery on triple antibiotic (Amp + Spm + Cm) agar plates either in the presence (+aTc) or absence (−aTc) of anhydrotetracycline induction with either of two different gRNAs targeting *bla* sequences (Supplementary Fig. 2a). Individual *E. coli* colonies carrying all three plasmids were then tested under an overnight growth protocol that imposes multi-generational antibiotic selection (see Supplementary Fig. 1 and Methods). In brief, colonies inoculated in Luria broth (LB) were grown overnight, maintaining antibiotic selection with or without aTc induction (Supplementary Fig. 1a). Although terminal culture densities were unaltered by Cas9 induction (Supplementary Fig. 2b), culture aliquots regrown under the more-stringent conditions imposed by selection on triple-antibiotic plates displayed significant Cas9-dependent reductions in CFU for both gRNA1 (~ 10-fold) and gRNA2 (~ 100-fold) (Fig. 1b). These differential responses to the Cas9 induction regimen are likely attributable to non-autonomous antibiotic rescue of mixed cells by secreted Bla in overnight liquid culture, absent during antibiotic plate selection of well isolated colonies. This strategy using CRISPR-Cas9 alone (e.g., without a homology template) is referred to hereafter as CRISPR-control.

As the targeting efficiency of gRNA2 under the aforementioned CRISPR-control framework matched previously reported reductions in CFU using a similar experimental paradigm^10^, we chose to test this gRNA in a “pro-active” configuration (Pro-AG) (Fig. 1c). We modified the Spm^R^ gRNA-expressing plasmid by flanking the gRNA2 expression cassette with *bla* sequence homology arms (HA, ~ 500 bp each, comparable in length to HA employed in eukaryotic systems) that directly abut the gRNA cleavage site, generating the pPro-AG plasmid (Supplementary Table 1). Since the gRNA cleavage site is absent from this plasmid, the encoded gRNA is unable to cleave these sequences in the presence of Cas9. Because standard *E. coli* strains do not support efficient homology-based insertion of genomic cassettes following induction of double-strand DNA breaks^13,14^, we made use of a cassette encoding recombinogenic lambda-Red (λRed)^15,16^ enzymes under control of an arabinose inducible promoter (arab) that is also encoded on this plasmid^9^. Plasmids pET (carrying the *bla* Amp^R^ target gene) and pCas9 were identical to those used for the “CRISPR- control” regimen (Fig. 1c). Following the parallel experimental scheme depicted in Supplementary Fig. 1b, we again observed no significant differences in CFU following initial transformation of the three Pro-AG plasmid components under triple-antibiotic (Amp + Spm + Cm) selection with or without Cas9 induction (i.e., ± aTc) (Supplementary Fig. 3a). Similarly, Cas9 ± λRed induction with aTc or arabinose, respectively, did not appreciably impact overnight culture densities under triple-antibiotic selection (Supplementary Fig. 3b).

### Pro-AG efficiently inactivates antibiotic resistance

As an initial comparison between the CRISPR-control (Fig. 1a) and the Pro-AG (Fig. 1c) configurations, overnight cultures of *E. coli* colonies transformed with the corresponding three-plasmid systems were grown in triple antibiotics with or without aTc (to induce Cas9), alone or in combination with arabinose (to induce λRed enzymes), then plated for CFU enumeration (Fig. 1d). In contrast to ~ 100-fold CFU reduction observed with the gRNA using our “CRISPR-control” component configuration (Fig. 1b, d), targeting of *bla* using the Pro-AG format (i.e., induction of Cas9 + λRed) led to a ~ 100,000-fold reduction in Amp^R^ CFU (Fig. 1d). For reasons that remain unclear, induction of λRed also yielded a modest reduction in CFU recovery in the CRISPR-control regimen; however, this effect was much more pronounced in a Pro-AG context (Fig. 1d, green vs. blue shading). The intensified reduction in Amp^R^ *E. coli* CFU could not be attributed to secondary effects of aTc and/or Cas9 induction, since no significant differences in CFU were observed under Amp selection (+ Amp) without Cas9 induction (–aTc) compared with Cas9 induction (+ aTc) without Amp selection (–Amp) (Supplementary Fig. 4a). Additional controls excluded suppressive effects of arabinose or λRed enzyme expression *per se*, as arabinose induction alone did not alter CFU recovery (Supplementary Fig. 4b).

We expected that either unrepaired CRISPR-mediated double-stranded DNA breaks or potential Pro-AG-mediated insertion of gRNA2 sequences into the corresponding cleavage site would inactivate the *bla* target gene, and therefore that *E. coli* undergoing such events would not be viable upon selection for Amp^R^. Indeed, among the few Amp^R^ colonies recovered following either CRISPR-control (green box) or Pro-AG (blue box) treatments (i.e., induction of Cas9 + λRed), sequence analysis showed that 100% (30/30) carried unaltered pET plasmids with intact *bla* gene-coding sequences at the gRNA target site (Fig. 1d, bottom panel). These rare examples of Amp^R^ *E. coli* colonies that evaded CRISPR or Pro-AG editing, likely from incomplete target cleavage of the high copy number plasmid, are referred to hereafter as “escapers”.

### Pro-AG results in precise homology-mediated editing

**Fig. 2:**
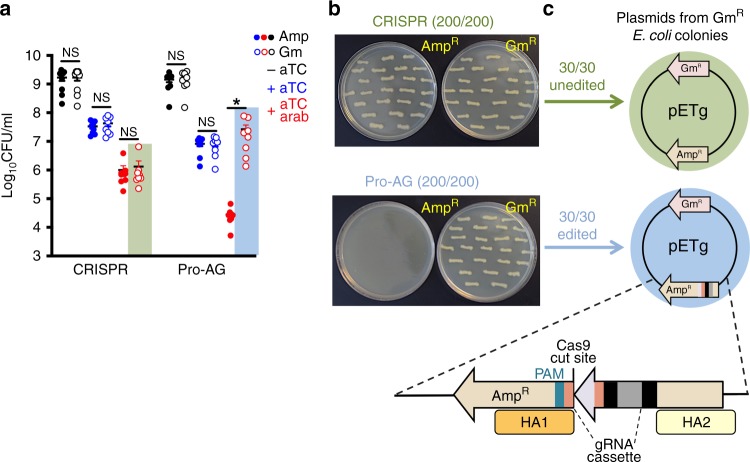
Efficient reduction of Amp^R^ CFU by Pro-AG results from homology-mediated insertion of active gRNA cassettes into the *bla* target gene. **a** Selection for *E. coli* CFU on ampicillin (Amp, filled dots) or gentamicin (Gm, open dots) plates following CRISPR-control- or Pro-AG-mediated targeting of the dual antibiotic-resistant target plasmid pETg (see pETg plasmid schematic in **c** from single colonies grown in the presence (+ aTc, blue dots), in the absence (−aTc, black dots) of anhydrotetracycline for induction of Cas9, or in combination of aTc and arabinose (+ aTc + arab, red dots) for induction of Cas9 and λRed, respectively. **b** Individual colonies isolated from gentamicin plates following Cas9 and λRed induction under CRISPR (green shaded box) or Pro-AG (blue shaded box) regimens in **a** were streaked on new ampicillin (Amp^R^, left images) and gentamicin (Gm^R^, right images) plates. Representative images of 200 colonies struck from CRISPR-control (top) or Pro-AG (bottom) are shown. **c** DNA sequence analysis of plasmids isolated from single colonies in **b** recovered from either the CRISPR-control regimen (Amp^R^ or Gm^R^ plates; green arrow) or the Pro-AG regimen (Gm^R^ plates; blue arrow). All 30 CRISPR-derivative clones analyzed revealed a fully intact pETg plasmid (unedited, green circle), whereas all 30 Pro-AG-derivative clones analyzed carried a perfect insertion of the homology-flanked gRNA2 expression cassette into the *bla* gene (zoom-in bottom scheme). The gRNA expression cassette is composed of the gRNA scaffold (purple), 20 bp gRNA-targeting sequences (pink and black), and the constitutive *tet* promoter (gray). Also indicated are the Cas9 cleavage site, and homology arms (HA1, dark yellow box and HA2, light yellow box) that flank the gRNA2 cleavage site in the *bla* target gene carried on the pETg plasmid. Data in **a** are plotted as the mean ± SEM, representing three independent experiments performed in triplicate and analyzed by Student’s *t* test. *N.S*. = not significant (*P* > 0.05) *P < 0.05. Source Data are available in the Source Data file.

In analogy to HDR-dependent copying of gene-drive elements in diploid organisms, enhanced gene targeting activity of Pro-AG over CRISPR-control (Fig. 1d) might reflect homology-mediated insertion of the gRNA cassette into the *bla* gRNA2/Cas9 cleavage site. We tested this hypothesis by incorporating a second selectable antibiotic resistance gene (gentamicin—Gm^R^) into the target pET plasmid (pETg; Supplementary Table 1), allowing recovery of *bla* gene-edited plasmids by selection on Gm plates (Supplementary Fig. 1). Consistent with high efficiency insertional copying, virtually the entire decrement of CFU seen in Pro-AG vs. CRISPR-control systems under Cas9 + λRed induction and Amp^R^ selection could be recovered under Gm^R^ selection (Fig. 2a, blue box). Likewise, as expected, all Pro-AG recovered Gm^R^ colonies failed to grow on Amp plates (Fig. 2b, bottom panel). In contrast to the nearly full restoration of the λRed-induced CFU decrement observed upon Gm^R^ selection with the Pro-AG regimen, no CFU recovery was observed under Gm^R^ vs. Amp^R^ selection using the CRISPR-control protocol for targeting *bla* (Fig. 2a, green box). A clear prediction of the Pro-AG hypothesis examined above is that all recovered Gm^R^ colonies should carry a precise insertion of the gRNA expression cassette. Indeed, analysis of 30 Gm-selected pETg plasmids from Pro-AG single colonies confirmed that the DNA cassette carried between the two homology arms, including the full gRNA2 scaffold and its promoter (Fig. 2c, bottom scheme), were perfectly copied from the donor plasmid (pPro-AGAmp, Fig. 1c; Supplementary Table 1) into the gRNA2 cleavage site of the *bla* target gene in all clones (Fig. 2c, blue circle). Thus the function of the targeted *bla* gene is disrupted by insertion of the gRNA expression cassette within its protein coding region. In contrast, all CRISPR-control-treated unedited *E. coli* escaper colonies recovered on Gm^R^ selection regrew under selection for Amp^R^ (Fig. 2b, top panel). Consistent with such escapers having evaded CRISPR-mediated mutagenesis, all (30/30) examined Amp^R^ + Gm^R^ CRISPR escapers displayed intact (unedited) *bla* gRNA2 target sites (Fig. 2c, green circle). In aggregate, the above findings support the hypothesis that the greatly enhanced reduction of Amp^R^ CFU observed upon Pro-AG versus CRISPR-control editing configurations is quantitatively attributable to homology-mediated insertion of gRNA2 sequences into *bla*-coding sequences on the target pETg plasmid. Of note, each insertional editing event expands the pool of functional gRNA donor scaffolds with extended homology arms in their newly copied plasmid context, which may initiate a chain reaction accelerating further insertional events.

As mentioned above, *E. coli* “escaper” colonies that evaded Pro-AG editing showed intact target sequences (Fig. 1d, bottom panel). We hypothesized that inefficient Cas9 cleavage in escaper cells could reflect competition with the bacterial DNA repair system. We tested this idea by comparing Pro-AG editing of the *bla* gene target carried by the pETg plasmid in WT vs. Δ*recA E. coli* (Supplementary Fig. 1b). A significant reduction of Amp^R^ CFUs was obtained in Δ*recA* cells, under Cas9 induction alone or in combination with λRed (Supplementary Fig. 5a). All target plasmids analyzed from both Gm^R^ WT and Δ*recA E. coli* colonies bore precise gRNA2 insertions into *bla* target gene (Supplementary Fig. 5b), paralleling the editing efficiencies observed in previous experiments (Fig. 2c). Although these findings indicate that RecA is not required for copying the gRNA2 expression cassette from donor plasmid to target gene, they also suggest that Pro-AG escaper cells are partially protected in a RecA-dependent manner. An additional previously observed mechanism of escape from CRISPR-Cas9-mediated editing of *E. coli* involves mutations in the gRNA-harboring plasmid^8,10^. Consistent with similar mechanisms operating in CRISPR-based editing experiments, analysis of the pPro-AG (Amp) plasmid from escaper cells revealed that ~ 50% of gRNA launching plasmids recovered from escaper lines harbored deletions spanning the operator region, the gRNA and/or the gRNA scaffold sequences (Supplementary Fig. 6a).

### Pro-AG depends on homology sequences flanking the gRNA

**Fig. 3:**
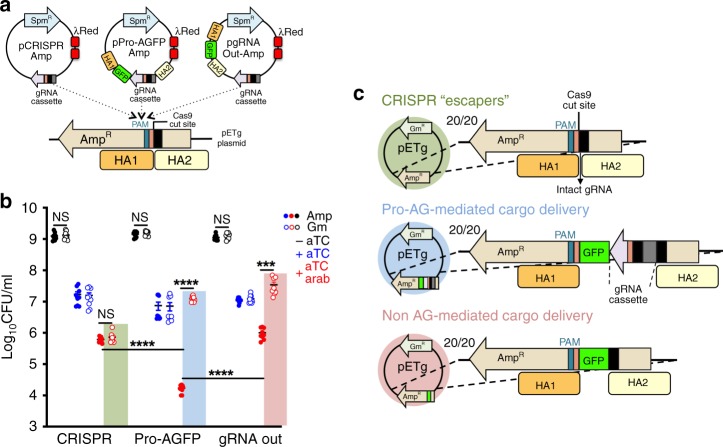
The Pro-AG system mediates efficient editing and cargo delivery dependent on precise flanking of the gRNA cassette by homology arms of the target gene. **a** Schematic of plasmids used to compare performance of CRISPR-control (pCRISPR Amp), Pro-AGFP (pPro-AGFPAmp: gRNA2 + GFP within homology arms), and external placement of gRNA2 outside of the homology arms flanked cassette (pgRNA Out-Amp: GFP-only within homology arms, gRNA outside of HA-cassette). **b** Recovery of *E. coli* CFU following CRISPR-control or Pro-AG using the three different plasmid configurations indicated in **a** and the various induction conditions indicated in the key. **c** Sequence analysis of targeted plasmids. pETg plasmids recovered from CRISPR-control-treated colonies (green circle) all display intact target sequence (“escapers”). Analysis of all Pro-AGFP-recovered pETg plasmids (blue circle) confirmed precise insertion of the gRNA + GFP cassette at the gRNA cut site, which consists of the full gRNA cassette (scaffold, purple; gRNA, pink and black, and *tet* promoter, gray) plus GFP. Although the gRNAOut-targeted pETg configuration (red circle) resulted in ~ 100-fold less efficient targeting of the *bla* target gene than the Pro-AGFP configuration, all plasmids isolated from colonies selected on Gm plates carried precise insertions of the GFP-only cassette. Data in **b** are plotted as the mean ± SEM, representing three independent experiments performed in triplicate and analyzed by Student’s *t* test. *N.S*. = not significant (*P* > 0.05); ***P < 0.001; ****P < 0.0001. Source Data are available in the Source Data file.

The above-described Pro-AG configuration, wherein the gRNA expression cassette is flanked directly by homology arms, represents the minimal possible self-copying element. We wondered if this system might also be exploited to deliver additional DNA cargo sequences efficiently. As a test case, a green fluorescence protein (GFP) transgene was included as cargo with the gRNA2 expression cassette between the *bla* gene homology arms (pPro-AGFPAmp, Fig. 3a, Supplementary Table 1). Using the same experimental design described above for targeting Amp^R^ + Gm^R^ plasmid pETg with the “gRNA-only” pPro-AG plasmid (Fig. 3a), the cargo-laden pPro-AGFP construct performed similarly to its minimal counterpart in reducing Amp^R^ CFU versus the CRISPR-control regimen (Fig. 3b). As for the minimal Pro-AG element, regrowth of single Pro-AGFP colonies recovered on Gm plates following Cas9 + λRed induction (Fig. 3b, blue box) revealed perfect insertion of the composite gRNA2:GFP cassette into the *bla* gRNA2 cleavage site in 100% of clones analyzed (Fig. 3c, blue circle). Again, escaper colonies recovered under the CRISPR-control regimen on Gm plates with Cas9 + λRed induction (Fig. 3b, green box) maintained pristine unedited gRNA target sequences and also displayed an Amp^R^ phenotype (Fig. 3c, green circle). We conclude that the Pro-AG system leads to highly efficient and precise insertion of the gRNA2:GFP cargo bearing cassette into the gRNA2 *bla* gene target site.

A defining feature of self-amplifying CRISPR-based gene-drive systems in diploid organisms is insertion of the gRNA-bearing cassette at the exact genomic site where the gRNA directs target cleavage^17^. If amplification of the gRNA gene dosage resulting from its being actively copied contributed to the enhanced efficiency of the Pro-AG system, then placement of the gRNA outside of the homology arm cassette (pgRNAOut-Amp) (Fig. 3a, Supplementary Table 1) would significantly reduce editing efficiency of the target plasmid. Indeed, the gRNA-Out configuration performed comparably to the CRIPSR-control protocol, leading only to an ~ 3 log_10_-fold reduction in CFU under Amp^R^ selection and Cas9 + λRed + induction (Fig. 3b). Thus, the entire boost in Amp^R^ CFU reduction provided by the Pro-AG system was abrogated by placing the gRNA outside of the homology arms, consistent with the hypothesis that the Pro-AG process acts via a positive feedback amplification mechanism (Fig. 3b). Although the gRNA-out configuration eliminated the ~ 2-log increment in CFU reduction observed with Pro-AGFP arrangement under Amp^R^ selection, significant CFU recovery was nonetheless observed upon Gm^R^ selection, suggesting that a large fraction of the baseline DNA breaks induced by CRISPR-control conditions were now being repaired by precise copying of the homology-flanked GFP-only cassette. Indeed, all 20 plasmids analyzed from Gm^R^ colonies under the gRNA-Out regimen (Fig. 3b, red bar) carried perfect insertions of the GFP-only cassette within the gRNA2 cleavage site of *bla* (Fig. 3c, red circle).

### Amplification of the gRNA contributes to Pro-AG performance

As copying of the gRNA2 expression cassette from a low (pPro-AG) to a high copy number plasmid (pETg) significantly amplified gRNA gene number and hence expression levels, we wondered whether the enhanced performance of Pro-AG versus CRISPR-control could be wholly attributable to this effect. We addressed this question by comparing Amp^R^ CFU reduction using CRISPR-control versus Pro-AG configurations in a situation where the *bla*-targeting gRNA2 was encoded from the outset on a high copy number plasmid, namely the pETg target plasmid itself, which would limit gRNA amplification to at most twofold. *E. coli* harboring pCas9 and pΔgRNA (Supplementary Table 1) expressing Cas9 and λRed, respectively, were transformed with either pETgCRISPR or pETgPro-AG plasmids (Supplementary Fig. 7a, Supplementary Table 1). Using our earlier CRISPR-control and Pro-AG-editing protocols under Amp selection (Supplementary Fig. 1), Pro-AG performed 1-logfold more efficiently than CRISPR-control in reducing Amp^R^ CFUs (Supplementary Fig. 7b). Although this Pro-AG gain is less than that achieved when gRNA2 was launched from a low copy plasmid, where we observed 2-logfold improved efficiencies, this more modest enhancement suggests that the full Pro-AG effect is not solely attributable to copy number amplification of gRNA2 (Fig. 1d). As before, significant CFU recovery occurred on Gm plates following Pro-AG editing (Supplementary Fig. 7b, top panel, blue shading), with perfect insertions of gRNA cassette into *bla*-coding sequences in 100% of pETgPro-AG plasmids analyzed (Supplementary Fig. 7b, bottom panel). We conclude that amplification of the homology-flanked gRNA, which may replicate itself ~ 50 times in a high copy plasmid, contributes significantly to the enhanced performance of Pro-AG. Additional mechanisms such as increased homology sequence length and double-strand DNA break repair may also contribute to enhanced Pro-AG efficiency compared with CRISPR only controls.

### Pro-AG acts via a self-amplifying mechanism

As yet another approach to assess the role of cassette amplification in the enhanced performance of Pro-AG versus CRISPR-control paradigms we made use of the temperature sensitive nature of replication of the low copy number gRNA2 donor plasmids. Switching overnight growth cultures from 30 °C (permissive temperature for plasmid replication) to 37 °C (non-permissive temperature) during the editing protocol reduced Spm^R^ CFU that harbor the gRNA2 plasmid by ~ 2.5-logfold (Supplementary Fig. 8a). As in previous experiments, Amp selection produced a significant reduction of Amp^R^ CFUs for both CRISPR-control and Pro-AG configurations following Cas9 and λRed induction (+ aTc + arab) at 30 °C, which was more pronounced for Pro-AG. In contrast, only the Pro-AG regimen, and not CRISPR-controls, also reduced Amp^R^ CFUs following Cas9 + λRed induction (+ aTc + arab) at 37 °C (Supplementary Fig. 8b). Based on these several independent lines of corroborating evidence, we conclude that incorporating the gRNA (± cargo) between homology arms to generate the active genetic cassette greatly increases targeting efficiency of a high copy number plasmid via a self-sustaining positive feedback loop.

### Pro-AG is amenable to manipulation of large plasmids

Large plasmids carried by various pathogens pose an important health problem and are challenging to manipulate by traditional methods. We wondered whether the enhanced Pro-AG system might again offer advantages in such a context. We chose an *E. coli* strain harboring the pCas9 plasmid and a ~ 50 Kb cosmid vector (Supercos SV305, Supplementary Table 1) carrying Amp and Km selection markers as a test case. As in previous experiments, cells were transformed with either the pCRISPR Amp control or pPro-AG (SuperCos) plasmids, the latter adopting a Pro-AG configuration to target the *bla* gene encoded on the cosmid (Supplementary Fig. 9a). Following our standard editing protocols (Supplementary Fig. 1), we observed a 1-logfold greater reduction in Amp^R^ CFUs with the Pro-AG regimen than CRISPR-control following Cas9 and λRed induction (+ aTc + arab) (Supplementary Fig. 9b, top panel). Indicative of Pro-AG-mediated double-strand DNA break repair, significant CFUs were rescued under Km selection (Supplementary Fig. 9b, top panel, blue shading box), and 100% of the Supercos SV305 cosmids isolated and sequenced showed a precise gRNA cassette insertion into *bla* (Supplementary Fig. 9b, bottom panel). Thus Pro-AG is well suited for gene editing large multi-copy plasmids, with efficient homology-mediated insertion of gRNA sequences into the targeted coding region.

### CRISPR and Pro-AG are equivalent for single locus editing

Given its efficiency in meeting the challenging task of targeting a high and moderate copy number plasmids, we asked whether the Pro-AG approach could similarly be employed to insert gRNA-only or gRNA + GFP cargo cassettes into a single-copy chromosomal target (e.g., the *lacZ* gene). We found that Pro-AG configurations indeed performed with high efficiency in this context as well (Supplementary Text and Supplementary Figs. 10 and 11). Because in this context there is no need for cassette amplification provided by Pro-AG, we predicted that targeting efficiency would not depend on the placement of the gRNA between homology arms. This expectation was confirmed by experiments in which the gRNA was placed between (gRNA-in) or outside (gRNA-out) of the GFP-containing cassette since both configurations performed equivalently in precise editing of the *lacZ* target gene (Supplementary Fig. 12).

## Discussion

Cumulatively, our results indicate that the Pro-AG configuration is 2–3 orders of magnitude more efficient in disrupting the activity of a high copy number target gene (*bla*) than the CRISPR-control arrangement, yielding to a 4–5 log_10_-fold reduction in Amp^R^ *E. coli*, fully attributed to precise insertion of the gRNA (± cargo) cassette from the editing vector into the targeted gRNA cleavage site. Increased potency of the Pro-AG versus CRISPR-control configuration in reducing antibiotic-resistant CFUs depends on the gRNA cassette being precisely flanked by *bla* homology arms, suggesting that an amplifying positive feedback loop occurs from increasing gRNA copy number, a potential rate-limiting component of the system. The high efficiencies of accurate Pro-AG cassette copying in bacteria are comparable to those attained by gene-drive systems in diploid organisms (> 90%)^1–3,18^. In addition to its high efficiency, Pro-AG maintains an ability to perform precise and potentially subtle edits of a target gene rather than simply eliminating the target sequence or bacteria carrying that locus.

Multiple genome-engineering applications could ultimately benefit from incorporation of the Pro-AG platform, including elimination of bacterial virulence factors carried by diverse episomal elements and resistance determinants in commensal bacteria as well as different prokaryotic pathogens, scrubbing antibiotic resistance genes from bacteria in the environment^19^, livestock, inland fish farms, or sewage treatment ponds^20^, reprogramming genetic circuits impacting bacterial physiology, or tailoring interactions among the microbiota in environmental or host niches. For each of these applications suitable delivery systems such as phage vectors^21^ or conjugative plasmids^22^ would need to be developed and tailored to the specific contexts in which they were being deployed.

## Methods

### Strains and culture conditions

*E. coli* strain MG1655 WT and ΔRecA were provided by the B. Palsson and Susan Lovett Laboratories, respectively. Liquid cultures of *E. coli* were grown in LB medium. When appropriate, antibiotics were added as following: chloramphenicol (Cm, 25 μg/ml), ampicillin (Amp, 100 μg/ml), spectinomycin (Spm, 50 μg/ml), and gentamicin (Gm, 10 μg/ml).

### Plasmid construction

All constructs used in this study are listed in Supplementary Table 1 with primer sequences provided in Supplementary Table 2. Plasmids pKDsgRNA (pCRISPR) and pCas9-CR4 (pCas9) (Table S1) were purchased from Addgene (Cambridge, MA). pCRISPR(AmpgRNA1), pCRISPR (AmpgRNA2) and pCRISPRlacZ were built as previously described^9^. The 20 bp targeting sequences of the gRNAs were cloned into pKDsgRNA using circular polymerase extension cloning (CPEC)^23^ of two linear PCR fragments (F1, 3 kb; F2, 4 kb). For pCRISPR(AmpgRNA1) (Supplementary Table 1), F1 and F2 were obtained using the paired primers 13_FwAmpgRNA1/14_RvF1 and 15_FwF2/16_RvAmpgRNA1 (Supplementary Table 2), respectively. For pCRISPR(AmpgRNA2) (Supplementary Table 1), F1 and 1 and 2 were obtained using the paired primers 17_FwAmpgRNA2 /14_RvF1 and 18_RvAmpgRNA2 /15_FwF2 (Supplementary Table 2), respectively. For pCRISPR*lacZ* (Supplementary Table 1), F1 and F2 were obtained using the paired primers 33_FwlacZgRNA/14_RvF1 and 34_RvlacZgRNA/15_FwF2 (Supplementary Table 2), respectively. PCR products with ~ 280 bp of overlapping homology sequences and 20 bp of overlap in the protospacer region were DpnI digested for at least 15 min then gel-purified. Fragments 1 and 2 were mixed together in equal amounts (200 ng each) and CPEC cloned with 15 cycles and Phusion High-Fidelity Polymerase (NEB). In all, 2.5 μl of the mixture was used to transform one-shot Stbl3 chemically competent *E. coli* cells (Thermo Fisher).

Plasmids expressing Pro-AG configurations pPro-AG(Amp), pPro-AG(*lacZ*), and pPro-AG Super-Cos (Supplementary Table 1) were built in two Gibson (NEBuilder, NEB) assembly steps using two linear PCR fragments with flanking overlapping sequences. For the pPro-AG(Amp) plasmid construct, the first step consisted of cloning *bla* sequence homology arm 1 in pCRISPR(AmpgRNA2) to generate the plasmid pCRISPR(AmpgRNA2 + HA1) (Supplementary Table 1) by amplification of two PCR fragments (F1 and F2) with paired primers 37_FwgRNA2/38_RvgRNA2 and 39_FwHA1Amp/40_RvHA1Amp (Supplementary Table 2), respectively. A second step consisted of cloning *bla* sequence homology arm 2 in pCRISPR(AmpgRNA2 + HA1) to generate plasmid pPro-AG(Amp) (Supplementary Table 1) by amplification of two PCR fragments (F1 and F2) with paired primers 41_FwgAmpHA1/42_RvgAmpHA1 and 43_FwHA2/44_RvHA2 (Supplementary Table 2), respectively. For the pPro-AG(*lacZ*) plasmid construct, a first step consisted of cloning *lacZ* sequence homology arm 1 in pCRISPR(*lacZ*) to generate pCRISPR(*lacZ* + HA1) plasmid (Supplementary Table 2) by amplification of two PCR fragments (F1 and F2) with paired primers 51_FwlacZgRNA/52_RvlacZgRNA and 53_FwHA1lacZ/54_RvHA1lacZ (Supplementary Table 2), respectively. A second step consisted of cloning *lacZ* sequence homology arm 2 in pCRISPR(*lacZ* + HA1) to generate plasmid pPro-AG(*lacZ*) (Supplementary Table 1) by amplification of two PCR fragments (F1 and F2) with paired primers 55_ FwlacZHA1/56_FwlacZHA1 and 57_FwlacZHA2/58_RvlacZHA2 (Supplementary Table 2), respectively. For the pPro-AG Super-Cos plasmid construct, we first cloned *bla* sequence homology arm 1 in pCRISPR(AmpgRNA2) to generate the plasmid pCRISPR(AmpgRNA2 + SV3B05HA1HA1) (Supplementary Table 1) by amplification of two PCR fragments (F1 and F2) with paired primers 106_FwHA1Supercos/107_RvHA1Supercos and 108_FwpKDsAmp2/109_RvpKDsAmp2 (Supplementary Table 2), respectively. A second step consisted of cloning *bla* sequence homology arm 2 in pCRISPR(AmpgRNA2 + SV3B05HA1HA1) to generate plasmid pPro-AG Super-Cos (Supplementary Table 1) by amplification of two PCR fragments (F1 and F2) with paired primers 110_Fw HA2Supercos/111_Rev HA2Supercos and Pr_112Fw CosHA1/Pr_113Rv CosHA1 (Supplementary Table 2), respectively.

Plasmids pETg, pPro-AGFP(Amp), pPro-AGFP(*lacZ*), pETgCRISPR, and pETgPro-AG (Supplementary Table 1) were generated by amplification of two linear PCR fragments (F1 and F2), with flanking overlapping sequences for Gibson assembly (NEBuilder, NEB). Paired primers 59_FwpET/60_RvpET and 61_FwGm/62_RvGm (Supplementary Table 2) were used to amplify PCR F1 and F2 to assemble pETg. Paired primers 69_FwP-AG(Amp)/73_RvP-AG(Amp) and 72_FwGFP-Amp/68_RvGFP-Amp (Supplementary Table 2) were used to amplify PCR F1 and F2 to assemble pPRO-AGFP(Amp). Paired primers 63_FwP-AG(lacZ)/64_RvP-AG(lacZ) and 65_FwGFP-lacZ/66_RvGFP-lacZ (Supplementary Table 2) were used to amplify PCR F1 and F2 to assemble pPro-AGFP(*lacZ*). Paired primers 98_FwProAG/99-RvPro-AG and 100_FwpETg(Pro-AG)/101_RvpETg(Pro-AG) were used to amplify PCR F1 and F2 to assemble pETgCRISPR. Paired primers 102_FwCRISPR/103-RvCRISPR and 104_FwpETg(CRISPR)/105_RvpETg(CRISPR) were used to amplify PCR F1 and F2 to assemble pETgCRISPR.

For the pgRNAout (Amp) and pgRNAout (*lacZ*) plasmid constructs, pCRISPR(AmpgRNA2) and pCRISPR(*lacZ*), respectively were linearized with the NcoI restriction enzyme. Subsequently, three fragments with flanking overlapping sequences (F1, F2, and F3) were PCR amplified as follows. For pgRNAout (Amp) construct, homology arms 1 (F1) and 2 (F2) from pPro-AG(Amp) and *gfp* (F3) from plasmid #48138 (Addgene), using primer pairs 67_FwHA1-GFPout/68_RVHA1-GFPout, 69_FwHA2-GFPout/70_RvHA2-GFPout and 71_FwGFP-GFPout/72_RvGFP-GFPout (Supplementary Table 2), respectively. For pgRNAout (*lacZ*) construct, homology arms 1 (F1) and 2 (F2) from pPro-AG(*lacZ*) and *gfp* (F3) from plasmid #48138 (Addgene), using primer pairs 88_FwHA1-lacZout/89_RvHA1-lacZout, 92_FwHA2-lacZout/93_RvHA2-lacZout and 90_FwGFP-lacZout/91_RvGFP-lacZout (Supplementary Table 2), respectively. Gibson assembly was carried out with the linearized vectors and the three corresponding overlapping PCR fragments to generate pgRNAout(Amp) and pgRNAout(*lacZ*) (Supplementary Table 1). All Gibson assembly reactions were transformed into NEB 5-alpha competent *E. coli* cells.

### *E. coli* transformation

Competent cells were prepared as previously described (Short Protocols in Molecular Biology, Chapter 1). For all plasmid transformations, 50 μl of aliquoted cells were gently thawed on ice, followed by addition of plasmid DNA prepared by QIAprep Spin Miniprep Kit (Qiagen), with 20 ng of each plasmid added to the transformation mix. *E. coli* cells were electroporated with the 1 mm Gene Pulser cuvette (Bio-Rad) at 1.6 kV and immediately resuspended in 250 μl super optimal broth with catabolite repression media. Cells were allowed to recover for 2 h at 30 °C (for cells transformed with pKDsgRNA derived plasmids) and for 1 h at 37 °C for cells transformed with pETg plasmid. Serial dilutions of cells were plated on LB with the corresponding antibiotic and they were incubated at 30 °C (48 h) or 37 °C (24 h), respectively.

### Induction of Cas9 and Lambda-Red enzymes

Single *E. coli* colonies obtained on plates after transformation were resuspended in 60 μl LB, which served as inoculum for 5 ml LB overnight cultures (~ 15 h) grown at 30 °C with shaking (200 rpm). When appropriate, Cas9 expression was induced in cells carrying the pCas9 plasmid (Supplementary Table 1) by adding 100 ng/ml anhydrotetracycline (aTc, Abcam) to the broth media. Similarly, when desired, λ-Red expression was induced in cells carrying pKdsgRNA derivative plasmids (Supplementary Table 1) by adding 50 mm arabinose (arab, Sigma) to the broth media during editing steps (Supplementary Fig. 1).

### *E. coli* colony counts

CFU were determined similarly to the miniaturized plating method described previously with small modifications. In brief, 25 μl of overnight culture, and dilution series of 10^−1^ to 10^−8^, were spotted in triplicate on LB plates containing the appropriate antibiotic selection and incubated overnight at 30 °C. No notable differences were observed when arab or aTc were added to the plates for λ-Red or Cas9 induction, respectively.

### Sequence analysis of editing events

For *bla-*editing events, pETg plasmids and Super-Cos (SV3B06) cosmids were purified from Amp^S^/Gm^R^ Amp^S^/Km^R^, respectively, single edited *E. coli* colonies by QIAprep Spin Miniprep Kit (Qiagen) and sequenced (Genewiz) with primer 35_FwAmp(ext) (Supplementary Table 2). pET-derivative plasmids from single escapers were analyzed following the same parameters. For *lacZ*-editing events, white *E. coli* colonies where quantified among the total white + blue mixed population of colonies on LB plates containing 1 mm IPTG and 0.03 % (v/v) Bluo-Gal (Teknova). Single white colonies were grown overnight at 37 °C and total genomic DNA isolated by DNeasy Blood and Tissue (Qiagen). PCR products were obtained with Q5 DNA polymerase (NEB) and using primer pairs 49_FwlacZseq and 50_RvlacZseq, followed by sequencing analysis (Genewiz) with primer 63_RvlacZint (Supplementary Table 2). Escaper blue colonies were also analyzed following the same parameters. gRNA plasmid constructs from escapers were sequenced with primers 27_Fw pKDSgRNAseq and 28_Rv pKDSgRNAseq.

### Visualization and quantification of GFP expression

In all, 10 μl aliquots from overnight cultures of *E. coli* exposed to the Pro-AGFP configuration in LB + 1 mm IPTG and under Cas9 and λ-Red induction were mounted onto slides with coverslips. GFP fluorescence was visualized using a Zeiss Axio Observer.D1 fluorescence microscope. For GFP fluorescence quantification, single white colonies from *E. coli* lacZ-editing plates were homogenized in 200 μl PBS and subsequently transferred to a 96-well plate. Optical density at 600 _nm_ and GFP fluorescence at 510 _nm_ were measured by using an EnSpire Plate Reader (PerkinElmer).

### Reporting summary

Further information on research design is available in the Nature Research Reporting Summary linked to this article.

## Supplementary information

Supplementary Information

Reporting Summary

## Data Availability

All data generated and analyzed during this study are included in the published article or provided in the Supplementary Information and are available from the corresponding author upon request. Source Data for Figs. 1–3 and Supplementary Figures 2–5, 7–12 are available in the Source Data file.

## Source Data

Source Data
