# Supplementary material for: A bacterial gene-drive system efficiently edits and inactivates a high copy number antibiotic resistance locus
Source: Nat Commun. 2019 Dec 16;10:5726. doi: 10.1038/s41467-019-13649-6 (PMC6915771; doi:10.1038/s41467-019-13649-6)
Supplement: Supplementary file 1 — Supplementary Information [file 41467_2019_13649_MOESM1_ESM.pdf]

Supplementary Information:

A bacterial gene-drive system efficiently edits and inactivates a high copy number antibiotic resistance locus

J. Andrés Valderrama[1,2], Surashree S. Kulkarni[1,3], Victor Nizet[1,2,4*], and Ethan Bier[1,3*]

Supplementary Text

Since including GFP cargo sequences in the Pro-AG system for insertion into a high copy number plasmid (Fig. 3b) had no obvious impact on the copying efficiency relative to that observed with the minimal gRNA-only cassette (Figs. 2b and 3b), we speculated that this system also might be adapted for inserting cargo into single-copy sequences in the bacterial chromosome. Such enhancement of currently available precise genome editing tools[18] could be useful for a wide variety of bacterial engineering applications. We constructed an analogous set of plasmids to those described above for evaluating CRISPR-control versus Pro-AG protocols in the context of editing the *lacZ* chromosomal target gene (Supplementary Fig. 10a). Comparing efficiencies of copying a minimal "gRNA-lacZ-only" versus a larger "gRNA-lacZ:GFP" cassette into the *lacZ* target site (Supplementary Fig. 10a, Supplementary Table 1), we followed a similar selection and induction procedure described above for the three plasmid strategy (Supplementary Fig. 1). In the context of the single-copy *lacZ* chromosomal target, induction of Cas9 following initial bacterial transformation resulted in a marked decrease in viable *E. coli* CFU following either the CRISPR-control or Pro-AG protocols (Supplementary Fig. 10b). These profound reductions in recovered CFU are consistent with previously reported findings on Cas9-induced cleavage of single copy genomic targets that presumably reflect the lethal effects of unrepaired chromosomal double-stranded breaks[19]. Similarly, overnight cultures seeded from single colonies achieved significantly lower cell densities following induction of Cas9 alone or together with λRed than observed without Cas9 induction (Supplementary Fig. 10c). Consistent with the lethal

DNA-break hypothesis, induction of the λRed DNA repair cassette together with Cas9 significantly increased CFU recovery **(**Supplementary Fig. 10d). Following induction of Cas9 alone, blue colonies comprised the overwhelmingly phenotype recovered on IPTG/X-Gal plates (intact *lacZ* gene function) (Supplementary Fig. 10e), suggesting that Cas9 had not acted on the target sequences in these escaper colonies. Indeed, all tested CRISPR and Pro-AG induced blue colony escapers recovered following induction of Cas9 alone carried fully intact *lacZ* target sequences (Supplementary Fig. 10a, bottom scheme). Induction of Cas9 + λRed applied to the CRISPR-control regimen only rarely produced white colonies (~2% of total colonies recovered, Supplementary Fig. 11a). In such white colonies, we were unable to amplify *lacZ* target sequences by PCR, perhaps reflecting large Cas9-induced deletion events (Supplementary Fig. 6b, top scheme), as observed in prior studies[20]. In contrast, induction of Cas9 + λRed in the Pro-AG and Pro-AGFP regimens led to recovery of ~90% white colonies, suggesting *lacZ* inactivation by precise insertion of the homology flanked DNA cassettes (Supplementary Fig. 6a). Indeed, all analyzed white colonies contained perfect gene-disrupting insertions of the gRNA-*lacZ*-only or gRNA-*lacZ*:GFP cargo cassettes into the gRNA-*lacZ* directed Cas9 cleavage site in the genomic *lacZ* locus (Supplementary Fig. 11b, middle and bottom schemes, respectively), mirroring prior results with the high copy plasmid. Because the GFP coding sequences were designed to be in-frame with *lacZ*, these Pro-AGFP edited bacteria also expressed the fluorescent GFP marker upon simultaneous induction of the *lac* operon with IPTG and activation of Cas9 + λRed (Supplementary Figs. 11c and d). We conclude that the Pro-AG system is well suited for high efficiency precision editing and delivery of DNA cargo into a chromosomal locus.

Sequencing of the gRNA constructs from Pro-AG escaper (blue) colonies following Cas9 + λRed induction revealed deletions in the *tet* operator region and sequence insertion downstream HA1 in 100% of clones analyzed (Supplementary Fig. 7b), suggesting escape dependent on inactivation of the gRNA donor plasmid.

Dosage amplification of gRNA2 accompanied its copying from its low-copy number launch plasmid to its high-copy plasmid targets, and experiments in which the gRNA component was either included in the amplifying cassette (gRNA-In) or left outside of the cassette (gRNA-Out) revealed that the enhanced efficiency of the Pro-AG system depended on the gRNA being placed within the homology arms cassette (Fig. 3a). In contrast, when targeting a single-copy *E. coli* chromosomal locus, we hypothesized that the absence of gRNA amplification in this context would translate to equal efficiencies of the gRNA-in and gRNA-out configurations. We compared Pro-AGFP (gRNA-In) versus gRNA-out configurations for insertion of the GFP cargo gene into *E. coli* chromosomal *lacZ* locus (Supplementary Fig. 12a) and observed that both configurations performed similarly, with no significant differences in CFUs recovered following parallel editing protocols (Supplementary Fig. 12b). Induction of Cas9 + λRed applied to gRNA-Out configuration yielded more >90% edited colonies (Supplementary Fig. 12c), with perfect insertion of GFP cargo into Cas9 cleavage site in the genomic *lacZ* locus at similar efficiency to the Pro-AGFP (gRNA-In) strategy (Supplementary Fig. 12d**)**.

Supplementary Figures

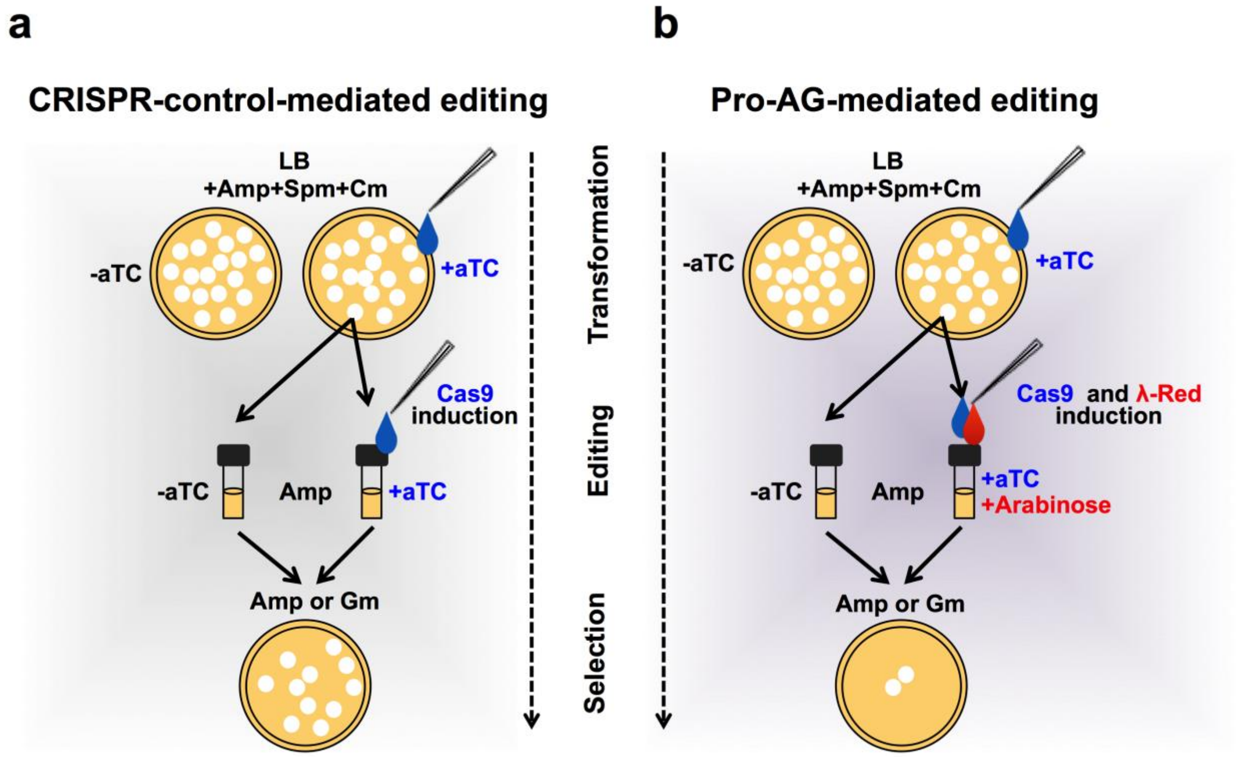

Supplementary Fig. 1 Pro-AG-mediated gene-editing and CRISPR-Control. Scheme of the method used for editing Amp resistance cassette from multi-copy plasmid through CRISPR-control **a** or Pro-AG **b** editing configurations *Escherichia coli* MG1655 cells were transformed with pET, pCas9 and pCRISPR or pPro-AG derivative plasmids in **a** or **b**, respectively. Cells were selected on LB plates containing triple antibiotic selection: ampicillin, spectinomycin and chloramphenicol (+Amp+Spm+Cm) in the presence or in the absence of anhydrotetracycline (aTc, Cas9 induction). Following transformation, single colonies were selected for an editing round, where bacteria was grown over night in LB broth in the presence of Amp and under induction of Cas9 with aTc. Additionally, in Pro-AG configuration **b**, recombinogenic λRed enzymes were induced with the addition of L-arabinose. Finally, aliquots were diluted and plated for selection and quantification of Amp or Gm resistant colonies in each editing configuration.

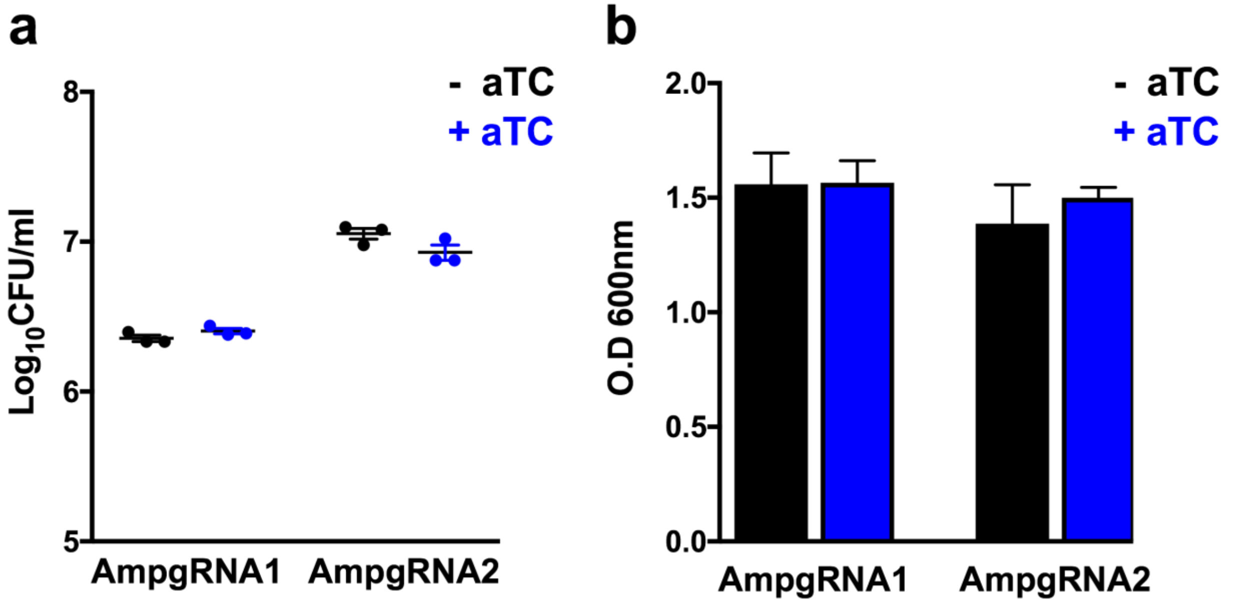

Supplementary Fig. 2 *Escherichia coli* MG1655 colony counts and cell density are not affected after transformation and plating with Cas9 switched off or on. **a** Quantification of Ampicillin resistant *E. coli* expressing CRISPR-control configuration that mediates Cas9 targeting of *bla* gene. Colony forming units (CFU) were quantified on ampicillin agar plates in the presence (+aTc, blue dots) or in the absence (-aTc, black dots) of the Cas9 inducer anhydrotetracycline. **b** Optical densities measured at 600 nanometers (O.D 600nm) from E. *coli* over-night cultures grown in the presence (+aTc, blue bars) or in the absence (-aTc, black bars) of anhydrotetracycline after CRISPR-control-mediated editing round AmpgRNA1 or AmpgRNA2 were expressed.

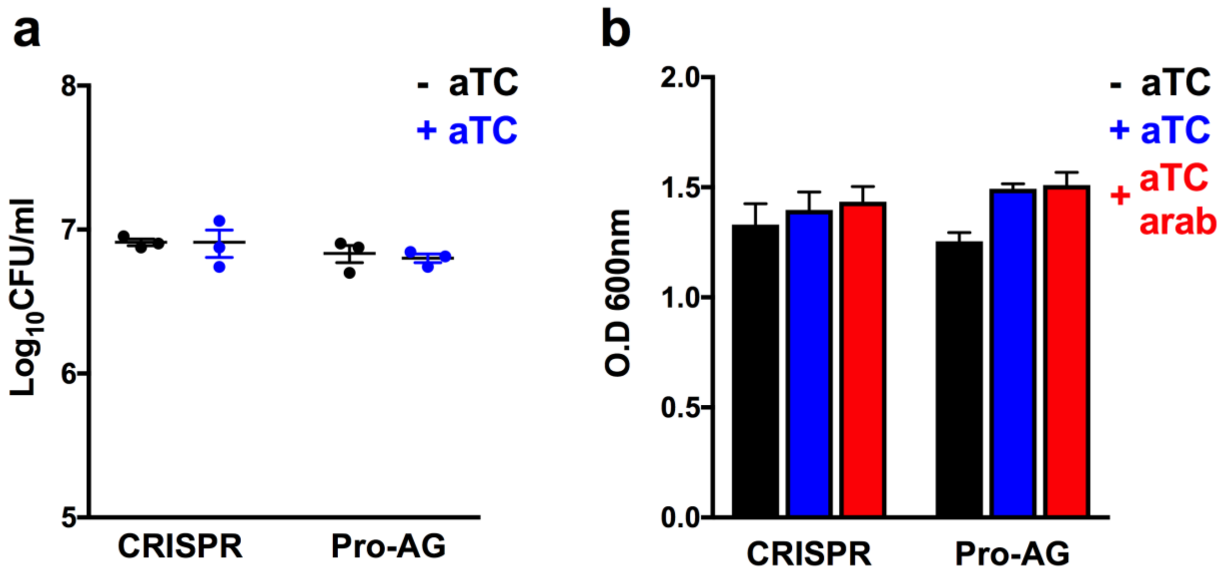

Supplementary Fig. 3 Number of *Escherichia coli* MG1655 colonies and cell density is not affected after transformation and overnight culture steps, respectively under CRISPR-control or Pro-AG editing configurations. **a** Quantification of Ampicillin resistant *E. coli* after transformation with the three plasmids system for CRISPR-control or Pro-AG editing configurations, that mediate Cas9 targeting of *bla* gene. Colony forming units (CFU) were quantified on LB ampicillin, spectinomycin and chloramphenicol agar plates and in the presence (+ aTc, blue dots) or in the absence (- aTc, black dots) of the Cas9 inducer anhydrotetracycline. **b** Optical densities (O.D.) measured at 600 nanometers from single *E. coli* colonies over-night cultures grown in the presence (+aTc, blue bars) or in the absence (-aTc, black bars) of anhydrotetracycline, and in combination of λRed enzymes induction with arabinose and Cas9 with aTc (+aTc +arab, red bars) after CRISPR-control or Pro-AG mediated editing rounds.

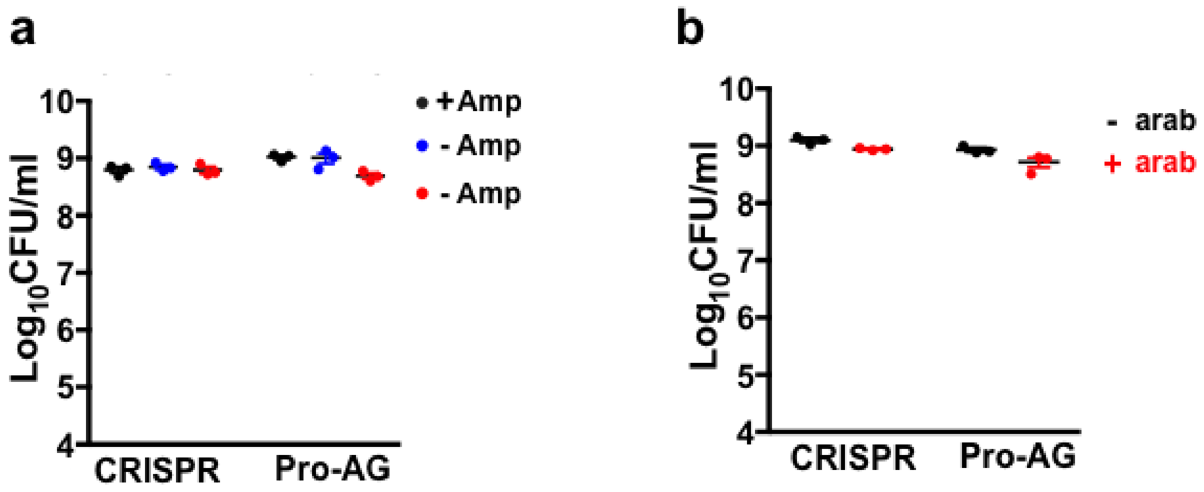

Supplementary Fig. 4 Cas9 and λRed induction with anhydrotetracycline and arabinose, respectively, do not affect *Escherichia coli* viability. **a** Quantification of *E. coli* colony forming units (CFU) on LB agar plates with (+Amp) or without (-Amp) after CRISPR-control or Pro-AG editing rounds when cells were grown in the presence (blue dots) or in the absence (black dots) of anhydrotetracycline (aTc) or in combination of aTc and arabinose (red dots). **b** Quantification of *E. coli* ampicillin resistant colony forming units (CFU) on LB ampicillin agar plates after CRISPR-control or Pro-AG editing rounds when cells were grown in the presence (+, red dots) or in the absence (-, black dots) of arabinose (arab), and under triple antibiotic selection, corresponding to the three plasmids used in our editing protocols.

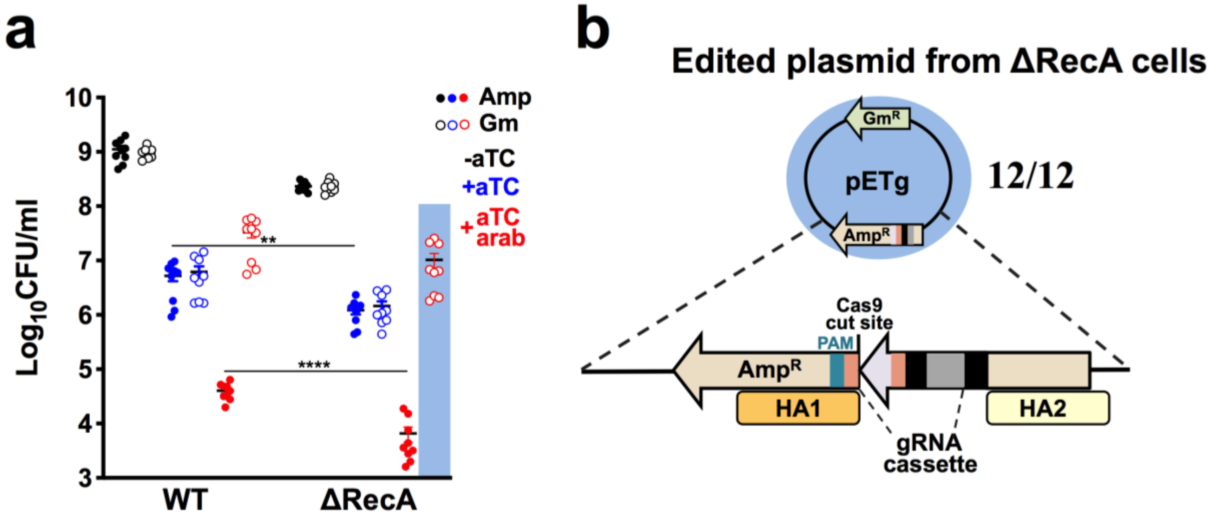

Supplementary Fig. 5 RecA is partially involved in the mechanism for *E. coli* escape from Pro-AG-mediated editing. **a** Comparison of CFU selection from wild type *E. coli MG1655* (WT) vs. MG1655 isogenic *recA* mutant (ΔRecA) strains on ampicillin (Amp, filled dots) or gentamicin (Gm, open dots) plates following Pro-AG-mediated targeting of the dual antibiotic resistant target plasmid pETg, from single colonies grown in the absence (-aTc, black dots), in the presence (+aTc, blue dots) of anhydrotetracycline for induction of Cas9 only, or in combination with arabinose for induction of both Cas9 and λRed (+aTc+arab, red dots). **b** DNA sequence analysis of plasmids isolated from single ΔRecA colonies in panel **a** recovered from the Pro-AG regimen (GmR plates; blue shaded box). All 12 Pro-AG-derivative clones analyzed carried a perfect insertion of the homology flanked gRNA2 expression cassette into the *bla* gene (zoom-in bottom scheme). The gRNA expression cassette is composed of the gRNA scaffold (purple), 20 bp gRNA targeting sequences (pink and black), and the constitutive *tet* promoter (grey). Also indicated are the Cas9 cleavage site, the protospacer adjacent motif (PAM), and homology arms (HA1, dark yellow box and HA2, light yellow box) that flank the gRNA2 cleavage site in the *bla* target gene carried on the pETg plasmid. Data in panel **a** are plotted as the mean ± s.e.m and analyzed by Student's t-test. **($P<0.01$); **** ($P<0.0001$).

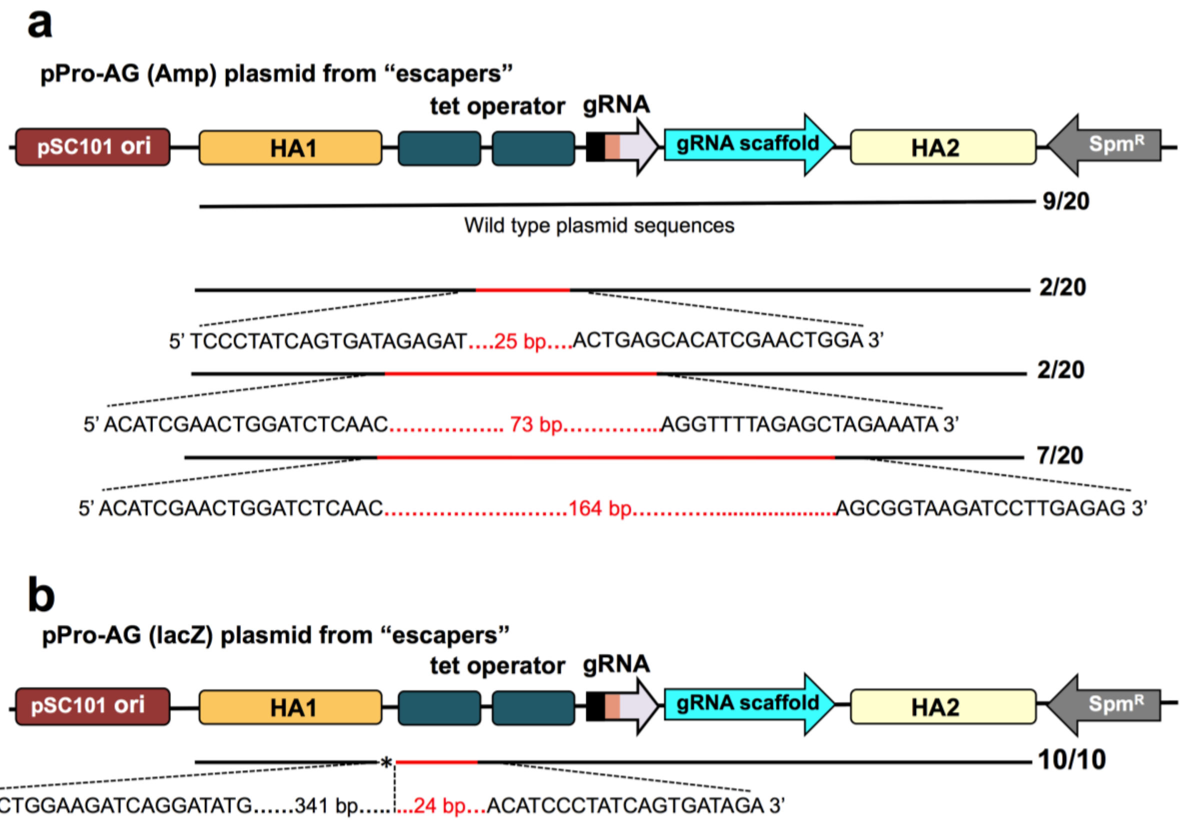

Supplementary Fig. 6 Sequence analysis of gRNA donor plasmids from Pro-AG escapers. Schematic of pPro-AG (Amp) **a** and pPro-AG (*lacZ)* **b** plasmids. Homology arms 1 (HA1, orange) and 2 (HA2, yellow) flanking *bla* **a** and *lacZ* **b** gRNAs, replication origin (pSC101ori, red), tet operator (cyan), gRNA (pink arrow), gRNA scaffold (turquoise arrow) and spectinomycin resistant gene (SpmR, grey arrow) are indicated. **a** Sequencing analysis of pPro-AG (Amp) plasmids from 20 escaper colonies of Pro-AG editing *bla* plasmid-encoded shows deletions (red lines) across the plasmid sequence in more than 50% of the plasmids. The remaining plasmids analyzed show intact wild type sequences (continuous black lines). **b** Sequencing analysis of pPro-AG (*lacZ*) plasmids from 10 escaper colonies of Pro-AG editing *chromosomal lacZ* gene shows similar pattern of *tet* operator deletion (red line) and sequence insertion (*). Length of insertion (discontinuous black line) and deletions (discontinuous red line) is indicated (bp). The 20 bp upstream and downstream specific insertions or deletions is also shown from the coding DNA strand (5'-3').

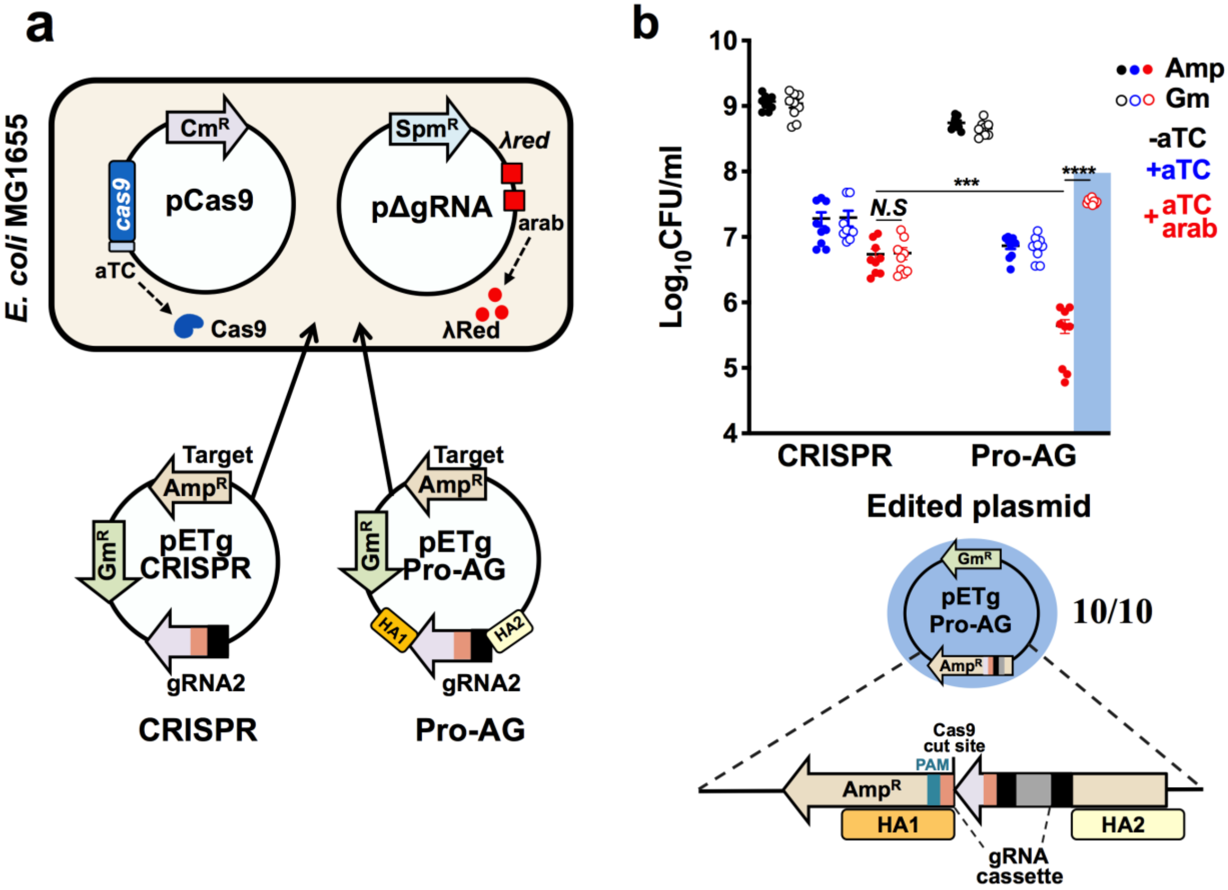

Supplementary Fig. 7 Differences in antibiotic resistance gene inactivation between Pro-AG and CRISPR-control configurations are partially dependent on gRNA levels. **a** Schematic of CRISPR-control and Pro-AG-mediated editing configurations of the beta-lactamase gene (*bla*) encoded on high-copy plasmids (pETg-CRISPR and pETg-Pro-AG) conferring resistance to ampicillin (AmpR, tan arrow) in *E. coli* MG1655. CRISPR-control configuration expresses gRNA2 targeting *bla* gene from the same pETgCRISPR target plasmid. Pro-AG configuration expresses the gRNA2 cassette flanked by *bla* (Amp*R*) homology arms (HA1 and HA2) that directly abut the gRNA2 cleavage site from the same pETg-Pro-AG target plasmid. pETg-CRISPR and pETg-Pro-AG plasmids also express a gentamicin resistance marker (GmR, green arrow). Cells were also transformed with a low copy plasmid, pCas9, maintained under chloramphenicol (CmR) selection, encodes Cas9 under control of an anhydrotetracycline (aTc) inducible promoter, and with a second low copy plasmid, pΔgRNA, maintained under spectinomycin (Spm) selection, encodes λRed under control of an arabinose (arab) inducible promoter. **b** Top panel. Selection for *E. coli* CFU on ampicillin (Amp, filled dots) or gentamicin (Gm, open dots) plates following CRISPR-control- or Pro-AG-mediated targeting of the dual antibiotic resistant target plasmids pETg-CRISPR and pETg-Pro-AG, respectively, from single colonies grown in the absence (-aTc, black dots), in the presence (+aTc, blue dots) of anhydrotetracycline for induction of Cas9 only, or in combination with arabinose for induction of both Cas9 and λRed (+aTc+arab, red dots). Bottom panel. DNA sequence analysis of plasmids isolated from single

colonies in top panel recovered from the Pro-AG regimen (Gm$_R$ plates; blue shaded box). All 10 Pro-AG-derivative clones analyzed carried plasmids target with a perfect insertion of the homology flanked gRNA2 expression cassette into the *bla* gene (zoom-in bottom scheme). The gRNA expression cassette is composed of the gRNA scaffold (purple), 20 bp gRNA targeting sequences (pink and black), and the constitutive *tet* promoter (grey). Also indicated are the Cas9 cleavage site, the protospacer adjacent motif (PAM), and homology arms (HA1, dark yellow box and HA2, light yellow box) that flank the gRNA2 cleavage site in the *bla* target gene carried on the pETg-Pro-AG plasmid. Data in panel **b** are plotted as the mean ± s.e.m and represent three independent experiments. Data analyzed by Student's t-test. *N.S*, not significant (*P*>0.05); ***(P<0.001); **** (P<0.0001).

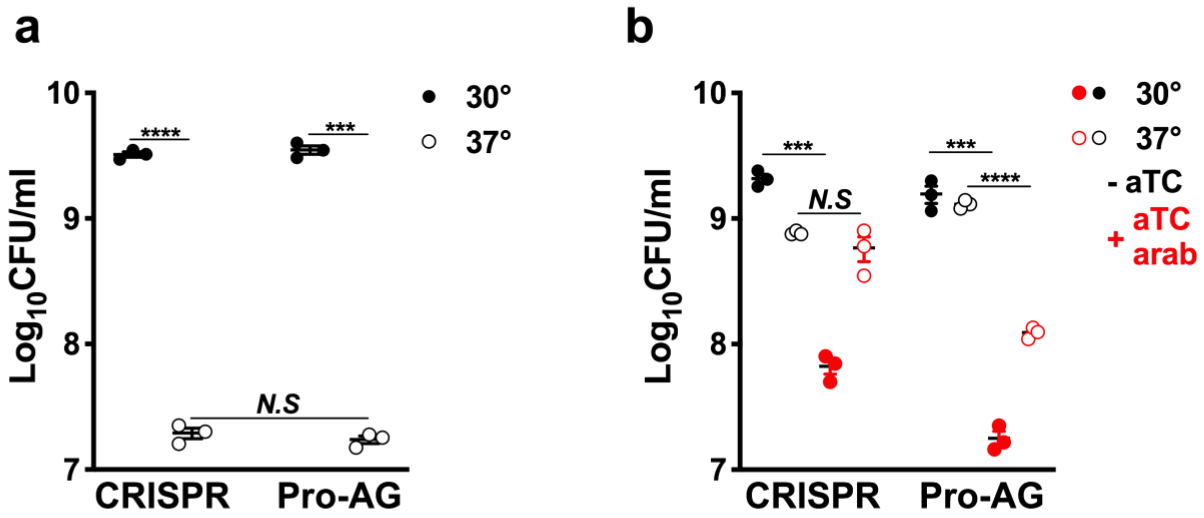

Supplementary Fig. 8 Pro-AG acts via a self-amplifying mechanism with a copied gRNA that remains functional on further editing events. **a** Recovery of spectinomycin resistant (SpmR) *E. coli* CFU from single CRISPR-control or Pro-AG edited colonies and following growth at 30°C (black dots) or 37°C (white dots). **b** Selection for *E. coli* CFU on ampicillin plates from single CRISPR-control or Pro-AG edited colonies and following growth at 30°C (filled dots) or 37°C (open dots) in the absence (-aTc, black dots) or in the presence of anhydrotetracycline in combination with arabinose (+aTc+arab, red dots). Bacteria growth at 30°C and 37°C conditions are permissive and non-permissive for gRNA-harboring plasmids replication, respectively. Data in panel **b** are plotted as the mean ± s.e.m and analyzed by Student's t-test. *N.S*, not significant (P>0.05); ***(P<0.001); **** (P<0.0001).

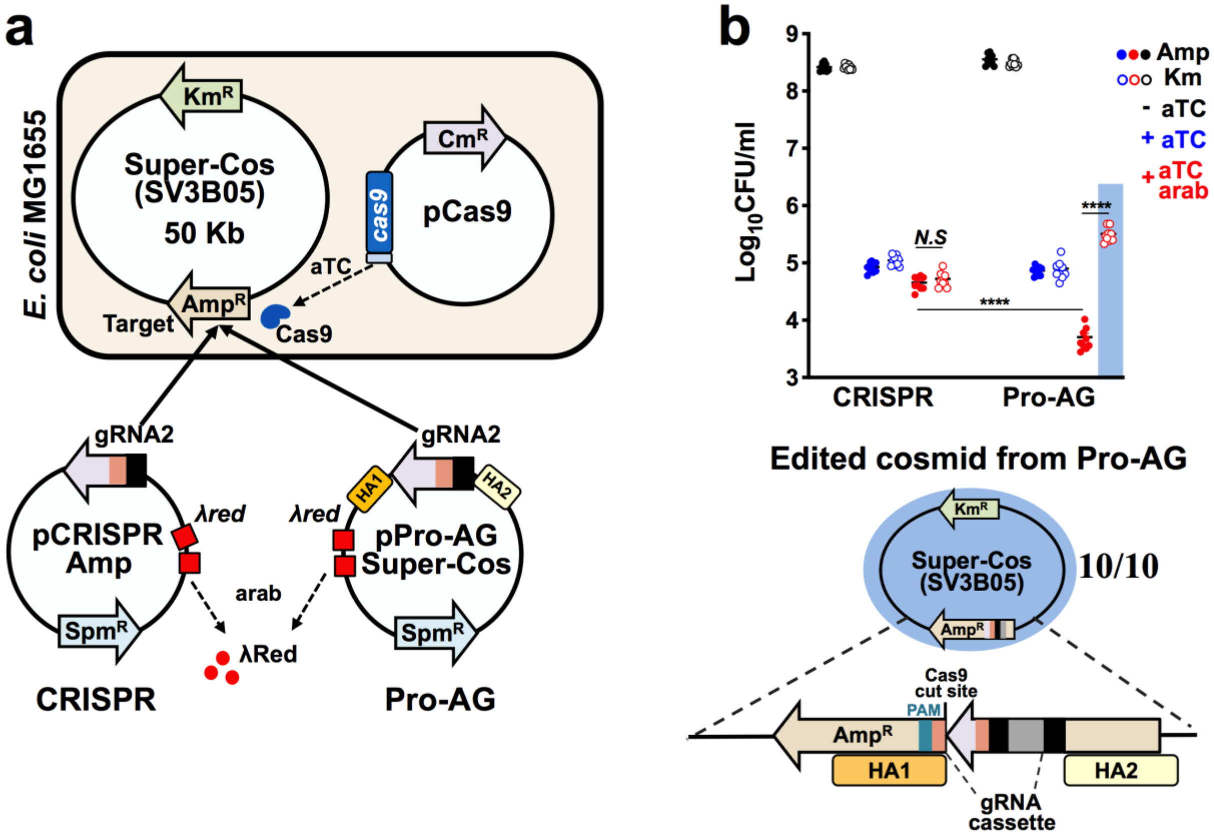

Supplementary Fig. 9 Pro-AG configuration is well suited for manipulation of large plasmids. **a** Schematic of CRISPR-control and Pro-AG-mediated configurations for inactivation of beta-lactamase gene (*bla*) encoded on long cosmid vector (Super-Cos SV3B05) conferring resistance to ampicillin (AmpR, tan arrow) in *E. coli* MG1655. Super-Cos SV3B05 cosmid also expresses a kanamycin resistance marker (KmR, green arrow). Cells were also transformed with a low copy plasmid, pCas9, maintained under chloramphenicol (CmR) selection, encodes Cas9 under control of an anhydrotetracycline (aTc) inducible promoter. CRISPR control configuration expresses gRNA2 targeting *bla* gene from pCRISPR Amp plasmid. Pro-AG configuration expresses the gRNA2 cassette flanked by *bla* (Amp*R*) homology arms (HA1 and HA2) that directly abut the gRNA2 cleavage site from pPro-AG Super-Cos plasmid. Also carried on plasmids pCRISPR Amp and pPro-AG Super-Cos are the recombinogenic λRed enzymes (λRed), which can be induced with L-arabinose (arab). **b** Top panel. Selection for *E. coli* CFU on ampicillin (Amp, filled dots) or kanamycin (km, open dots) plates following CRISPR-control- or Pro-AG-mediated targeting of the dual antibiotic resistant target cosmid Super-Cos SV3B05, from single colonies grown in the absence (-aTc, black dots), in the presence (+aTc, blue dots) of anhydrotetracycline for induction of Cas9 only, or in combination with arabinose for induction of Cas9 and λRed (+aTc+arab, red dots). Bottom panel. DNA sequence analysis of plasmids isolated from single colonies in top panel recovered from the Pro-AG regimen (KmR plates; blue shaded box). All 10 Pro-AG-derivative clones analyzed carried a perfect insertion of the homology flanked gRNA2 expression cassette into the *bla* gene (zoom-in bottom scheme). The gRNA expression cassette is composed of the gRNA scaffold (purple), 20

bp gRNA targeting sequences (pink and black), and the constitutive *tet* promoter (grey). Also indicated are the Cas9 cleavage site, the protospacer adjacent motif (PAM), and homology arms (HA1, dark yellow box and HA2, light yellow box) that flank the gRNA2 cleavage site in the *bla* target gene carried on the Super-Cos SV3B05 cosmid. Data in panel **b** are plotted as the mean ± s.e.m and analyzed by Student's t-test. *N.S*, not significant (P>0.05); **** (P<0.0001).

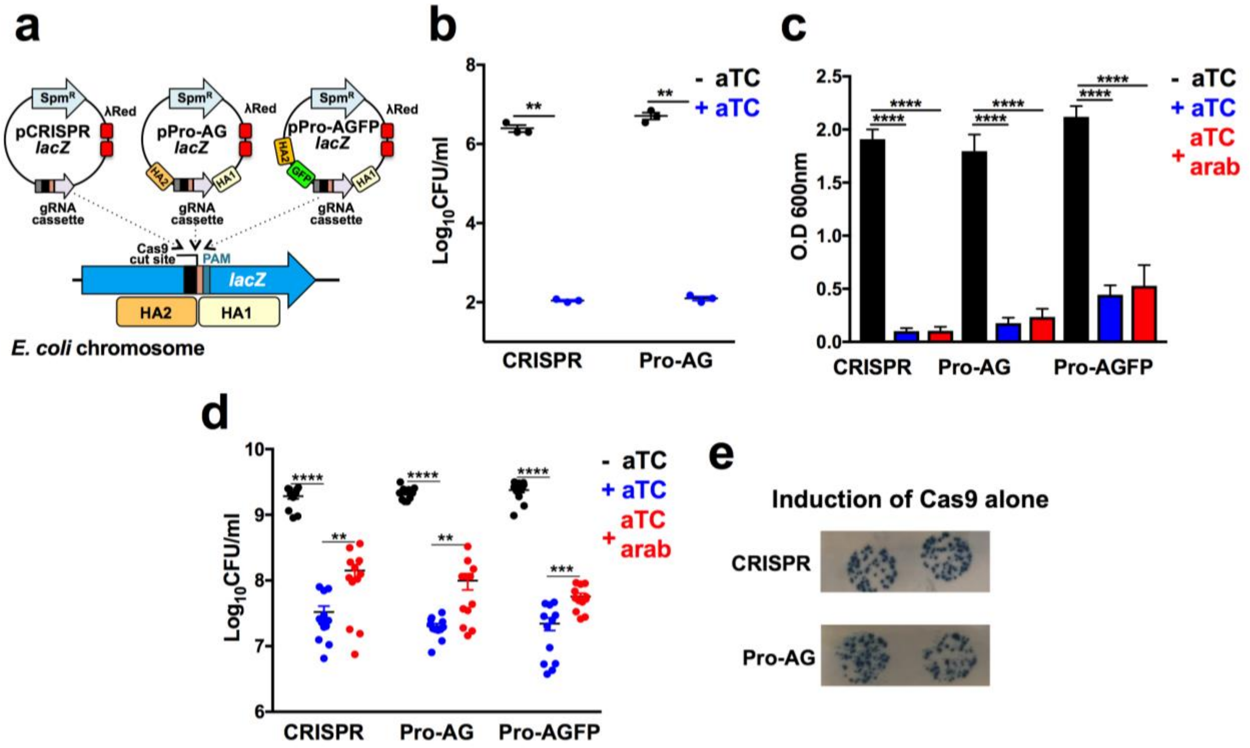

Supplementary Fig. 10 Comparison between CRISPR-control and Pro-AG editing configurations when targeting chromosomal *Escherichia coli lacZ* gene. **a** Schematic of the three plasmids used for editing the genomic *lacZ* locus: CRISPR-control (pCRISPR*lacZ*); gRNA-only or Pro-AG (pPro-AG*lacZ*); and gRNA + GFP or Pro-AGFP (pPro-AGFP*lacZ*). Precise insertion of homology flanked cassettes would inactivate the *lacZ* chromosomal *E. coli* locus (*lacZ*, blue arrow) and in the case of the pPro-AGFP*lacZ* construct, result in in-frame fusion with GFP. **b** Quantification of *E. coli* colony forming units (CFU) following initial transformation with the corresponding CRISPR-control or Pro-AG editing configuration plasmids. CFUs were quantified on LB spectinomycin and chloramphenicol agar plates and in the presence (+aTc, blue dots) or in the absence (-aTc, black dots) of the Cas9 inducer anhydrotetracycline. **c** Optical densities measured at 600 nanometers (O.D 600nm) from single *E. coli* colonies after transformation and over night cultures when cells were grown in the absence (-aTc, black bars), or presence of anhydrotetracycline (+aTc, blue bars) or in combination of aTc and arabinose (+aTc+arab, red bars) compared under the following configurations: CRISPR, Pro-AG, or Pro-AGFP. **d** Quantification of *E. coli* colony forming units (CFU) after CRISPR-control, Pro-AG and Pro-AGFP mediated editing of *lacZ* loci. Cells were grown with (+aTc, blue dots) or without (-aTc, black dots) Cas9 induction with anhydrotetracycline, or in combination of Cas9 and λRed induction with aTc and arabinose, respectively (+aTc +arab, red dots). **e** Appearance of dilutions spots carrying isolated colonies obtained under CRISPR-control (top) versus Pro-AG (bottom) configurations and after induction of Cas9 only. Data in panels **b**, **c** and **d** are plotted as the mean ± s.e.m and represent three independent experiments. Data analyzed by Student's t-test. ** (P<0.01); **** (P<0.0001).

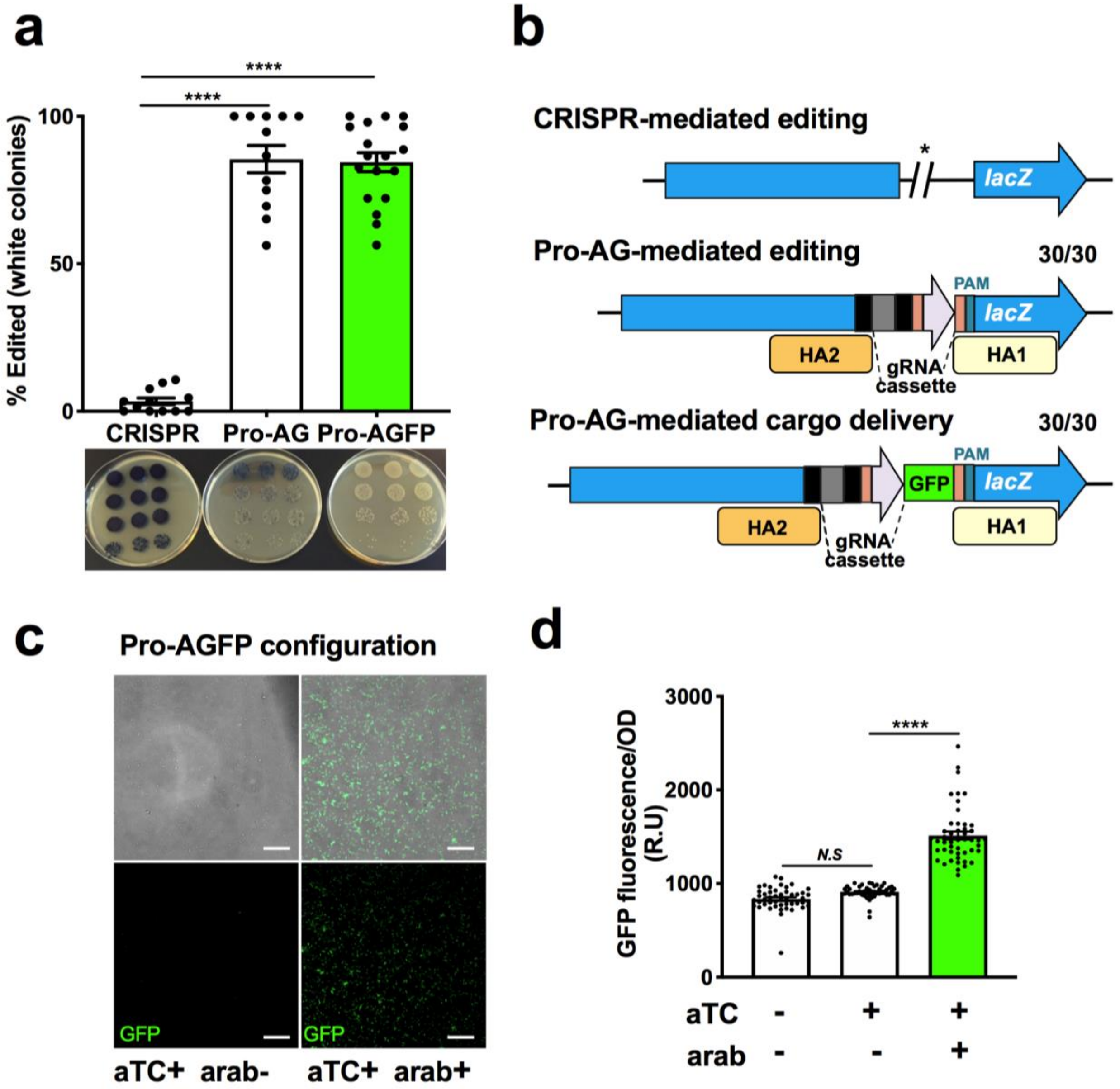

Supplementary Fig. 11 The Pro-AG configuration mediates enhanced editing of chromosomal targets. **a** Quantification of *E. coli lacZ* loci editing or mutagenic events (% white colonies of total colonies recovered), on plates-containing IPTG and X-Gal and after treatments with CRISPR-control, Pro-AG, or Pro-AGFP systems (top panel). Representative images of IPTG/X-Gal plates containing unedited (blue colonies) or edited (white colonies) are shown for the corresponding editing configuration (bottom panel). **b** Sequence analysis of edited *lacZ* genomic targets. CRISPR-control edited colonies displayed large chromosomal deletions (*) of the *lacZ* locus (top scheme), which were not analyzed further. Pro-AG events all resulted in the precise insertion of full *lacZ*-gRNA cassette within the gRNA target site in *lacZ* (middle scheme), and all Pro-AGFP events contained perfect insertions of the gRNA+GFP cassette (bottom scheme) resulting in in-frame fusion of the *lacZ* and *gfp* genes. **c** Fluorescence microscopy images from *E. coli* over-night cultures expressing Pro-AGFP configuration and growing under Cas9 only (left panels, aTc+ arab-), or in combination of Cas9 and

λRed (right panels, aTc+ arab+) activation conditions (+aTc+arab) and in the presence of the *lac* operon inducer molecule IPTG. GFP (bottom panels, green) and merged fluorescence and phase-contrast images (top panels) are shown. Scale bars (20 μm). **d** Quantification of GFP fluorescence from *E. coli* single colonies after Pro-AGFP configuration treatment in the absence (-) or in the presence (+) of Cas9 (aTc) and λRed (arab) activation conditions. The Y-axis quantifies the ratio of absorbance at 525nm/600nm and is indicated as GFP fluorescence/OD in relative units (RU). Data in panels **a** and **d** were plotted as the mean ± s.e.m and represent five independent experiments and 50 single colonies from each condition, respectively. Data analyzed by Student's t-test. *N.S*, not significant (P>0.05); **** (P<0.0001).

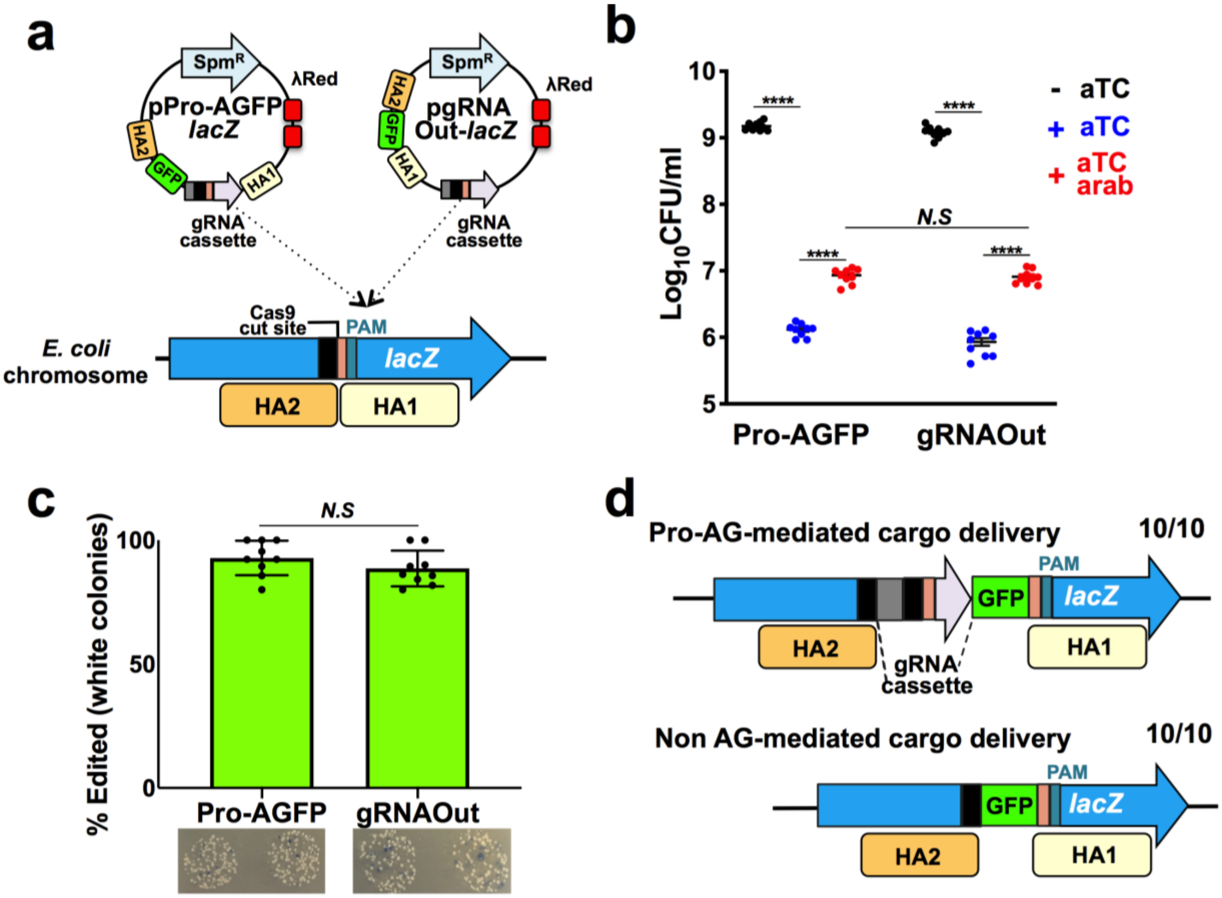

Supplementary Fig. 12 Effect of gRNA placement on targeting the chromosomal *lacZ* gene. **a** Schematic of plasmids used to compare performance of Pro-AGFP (pPro-AGFP *lacZ*: *lacZ*gRNA+GFP within homology arms), and external placement of l*acZ*gRNA outside of the homology arms flanked cassette (pgRNAOut-*lacZ*; GFP-only within homology arms, gRNA outside of HA-cassette. **b** Quantification of *E. coli* colony forming units (CFU) after, Pro-AGFP and gRNA-Out mediated editing of *lacZ* loci. Cells were grown with (+aTc, blue dots) or without (-aTc, black dots) Cas9 induction with anhydrotetracycline, or in combination of Cas9 and λRed induction with aTc and arabinose, respectively (+aTc +arab, red dots). **c** Quantification of *E. coli lacZ* loci editing or mutagenic events (% white colonies of total colonies recovered), on plates-containing IPTG and X-Gal and after treatments with Pro-AGFP and gRNA-Out configurations (top panel). Representative images of IPTG/X-Gal plates containing unedited (blue colonies) or edited (white colonies) are shown for the corresponding editing configuration (bottom panel). **d** Sequence analysis of edited *lacZ* genomic targets. All Pro-AGFP events contained perfect insertions of the gRNA+GFP cassette (top scheme). All gRNA-Out events contained perfect insertions of the GFP-only cassette (bottom scheme). Data in panels **b** are plotted as the mean ± s.e.m and analyzed by Student's t-test. *N.S*, not significant (*P*>0.05); **** (P<0.0001).

Supplementary Table 1. Plasmids used in this study

Plasmid	Relevant genotype or phenotype[α]	Source
pKDsgRNA	Spm_r, *ori*pSC101, low copy number vector expressing the gRNA scaffold from the *tet* promoter and λ-*red* from the *araBAD* promoter	Reference 9
pCas9-CR4 or pCas9	Cm_r, *ori*p15A, low copy number vector expressing *cas9* from the *tet* promoter	Reference 9
pET-Duet1	Amp_r, *ori*F1, *lacI*$_+$, *lacZ*α, T7 promoter, *lac* operator, multi copy cloning and expression vector.	(Novogen)
pETg	Amp_r, Gm_r, pET-Duet1 derivative, expressing Gm_r cassette from the pGm promoter	This work
pCRISPR(AmpgRNA1)	Spm_r, pKDsgRNA derivative expressing AmpgRNA1 from the *tet* promoter; carries 20 nucleotides (5' TTACTTCTGACAACGATCGG3 ') to direct Cas9 cleavage of Amp_r cassette in pET and/or pET-g plasmid targets	This work
pCRISPR(AmpgRNA2)	Spm_r, pKDsgRNA derivative, expressing AmpgRNA2 from the *tet* promoter; carries 20 nucleotides (5' ATCGAACTGGATCTCAACAG 3') to direct Cas9 cleavage of Amp_r cassette in pET and/or pET-G	This work
pCRISPR(AmpgRNA2+HA1)	Spm_r, pCRISPR(AmpgRNA2) derivative; carries *bla* sequence homology arm 1 upstream AmpgRNA2 cassette	This work
pPro-AG(Amp)	Spm_r, pCRISPR(AmpgRNA2+HA1) derivative; carries AmpgRNA2 cassette flanked by *bla* sequence homology arms 1 and 2	This work
pPro-AGFP(Amp)	Spm_r, pPRO-AG derivative; carries AmpgRNA2 cassette flanked by *bla* sequence homology arms and *gfp* sequence flanked by homology arm 1 and AmpgRNA2.	This work
pgRNAout (Amp)	Spm_r, pCRISPR(AmpgRNA2) derivative, carries *gfp* sequence flanked by *bla* sequence homology arms (500 pb each) in a different location to the one for AmpgRNA2 cassette	This work
pCRISPR(*lacZ*)	Spm_r, pKDsgRNA derivative expressing *lacZ*gRNA from the *tet* promoter; carries 20 nucleotides (5' TGGAAGATCAGGATATGTGG3 ') to direct Cas9 cleavage of *lacZ* in the *E. coli* MG1655 chromosome.	This work
pCRISPR(*lacZ*+HA1)	Spm_r, pCRISPR(*lacZ*) derivative, carries *lacZ* sequence homology arm1 upstream *lacZ*gRNA cassette	This work
pPro-AG(*lacZ*)	Spm_r, pCRISPR(*lacZ*+HA1) derivative; carries *lacZ*gRNA cassette flanked by *lacZ* sequence homology arms 1 and 2	This work
pPro-AGFP(*lacZ*)	Spm_r, pPRO-AG derivative; carries l*acZ*gRNA cassette flanked by *lacZ* sequence homology arms and *gfp* sequence flanked by homology arm 1 and l*acZ*gRNA	This work
pETgCRISPR	Amp_r, Gm_r, pETg derivative; carries AmpgRNA2 cassette	This work
pETgPro-AG	Amp_r, Gm_r, pETg derivative; carries AmpgRNA2 cassette flanked by *bla* sequence homology arms 1 and 2	This work
pCRISPR (AmpgRNA2+SV3B05HA1)	Spm_r, pCRISPR(AmpgRNA2) derivative; carries *bla* sequence homology arm 1 of SV3B05 upstream AmpgRNA2 cassette	This work
Super-Cos (SV3B06)	Amp_r, Km_r, SuperCos derivative (Agilent); carries 40,647 bp from *Streptomyces venezuelae*	N. Tschowri Lab
pPro-AG Super-Cos	Spm_r, pCRISPR(AmpgRNA2+SV3B05HA1) derivative; carries AmpgRNA2 cassette flanked by *bla* sequence homology arms 1 and 2 of SV3B05	This work
pgRNAout (*lacZ*)	Spm_r, pCRISPR(*lacZ*) derivative, carries *gfp* sequence flanked by *lacZ* sequence homology arms (500 pb each) in a different location to the one for *lacZ*gRNA cassette	This work

Supplementary Table 2. Oligonucleotides used in this study

Primer ID	Sequence 5'-3'	Use
59_FwpET	aaactcccacatggattcgaaatcaatctaaagtatatatgagtaaacttggtctgacag	Amplification of 5458 bp (F1), spanning the pET vector, used for Gibson assembly of pETG.
60_RvpET	cccaagtaccgccacctaaaaaacttcatttttaatttaaaaggatctaggtgaagatcc	
61_FwGm	taaattaaaaatgaagtttttaggtggcggtacttggg	Amplification of 656 bp (F2), spanning Gm cassette, used for Gibson assembly of pETG
62_RvGm	tatatactttagattgatttcgaatccatgtgggagtttattcttgac	
13_FwAmpgRNA1	ttacttctgacaacgatcgggttttagagctagaaatagcaag	Amplification of 2868 bp (F1) to construct pCRISPR(AmpgRNA1)
14_RvF1	tttataacctccttagagctcga	
15_FwF2	ccaattgtccatattgcatca	Amplification of 4434 bp (F2) to construct pCRISPR(AmpgRNA1)
16_RvAmpgRNA1	ccgatcgttgtcagaagtaagtgctcagtatctctatcactga	
17_FwAmpgRNA2	atcgaactggatctcaacaggttttagagctagaaatagcaag	Paired with primer 14 for amplification of 2868 bp (F1) to construct pCRISPR(AmpgRNA2)
18_RvAmpgRNA2	ctgttgagatccagttcgatgtgctcagtatctctatcactga	Paired with primer 15 for amplification of 4434 bp (F2) to construct pCRISPR(AmpgRNA2)
37_FwgRNA2	tacatcgaactggatctcaacttccctatcagtgatagagattgacat	Amplification of 7002 bp (F1), spanning pCRISPR(AmpgRNA2) for Gibson assembly of pCRISPR(AmpgRNA2+HA1)
38_RvgRNA2	agccgtagttaggccaccacaattccagaaatcatccttagcgaaagc	
39_FwHA1Amp	taaggatgatttctggaattgtggtggcctaactacggc	Amplification of *bla* sequence HA1 (540 bp, F2) for Gibson assembly of pCRISPRgRNA2+HA1
40_RvHA1Amp	ctctatcactgatagggaagttgagatccagttcgatgtaacccact	
41_FwgAmpHA1	ggcggataaagttgcaggacttgggcccgaacaaaaact	Amplification of 7502 bp (F1), spanning pCRISPR(gRNA2+HA1) for Gibson assembly of pPro-AG(Amp)
42_RvgAmpHA1	tctcaaggatcttaccgctggcttcaaaaaaagcaccgactcg	
43_FwHA2	gtcggtgctttttttgaagccagcggtaagatccttgagagttt	Amplification of *bla* sequence HA2 (540 bp, F2) for Gibson assembly of pPro-AG(Amp)
44_RvHA2	gagtttttgttcgggcccaagtcctgcaactttatccgcct	
69_FwP-AG(Amp)	tggacgagctgtacaagtaacttccctatcagtgatagagattgaca	Amplification of 8001 bp (F1), spanning pCRISPR(gRNA2+HA1) for Gibson assembly of pPro-AGFP(Amp)
73_RvP-AG(Amp)	cctcgcccttgctcaccatttgagatccagttcgatgtaacccact	
72_FwGFP-Amp	tacatcgaactggatctcaaatggtgagcaagggcgag	Amplification of *gfp* sequence (760 bp, F2) for Gibson assembly of pPro-AGFP(Amp)
68_RvGFP-Amp	ctctatcactgatagggaagttacttgtacagctcgtccatgccg	
33_FwLacZgRNA	tggaagatcaggatatgtgggttttagagctagaaatagcaag	Paired with primer 14 for amplification of 2868 bp (F1) to construct pCRISPR(*lacZ*)
34_RvLacZgRNA	ccacatatcctgatcttccagtgctcagtatctctatcactga	Paired with primer 15 for amplification of 4434 bp (F2) to construct pCRISPR(*lacZ*)
51_FwlacZgRNA	atctggaagatcaggatatgcttccctatcagtgatagagattgacatcc	Amplification of 7002 bp (F1), spanning pCRISPR(*lacZ*gRNA) for Gibson assembly of pCRISPR(*lacZ*gRNA+HA1)
52_RvlacZgRNA	ggcctcttcgctattacgccaattccagaaatcatccttagcgaaagc	
53_ FwHA1lacZ	taaggatgatttctggaattggcgtaatagcgaagaggcc	Amplification of *lacZ* sequence HA1 (540 bp, F2) for Gibson assembly of pCRISPR(*lacZ*gRNA+HA1)
54_ RvHA1lacZ	ctctatcactgatagggaagcatatcctgatcttccagataactgccg	
55_ FwlacZHA1	atggtcaggtcatggatgagttgggcccgaacaaaaactcatctcag	Amplification of 7502 bp spanning pCRISPR(lacZgRNA+HA1) for Gibson assembly of pPro-AG(*lacZ*)
56_ FwlacZHA1	aaaatgccgctcatccgccagcttcaaaaaaagcaccgact	
57_FwlacZHA2	gtcggtgctttttttgaagctggcggatgagcggca	Amplification of *lacZ* sequence HA2 (541 bp, F2) for Gibson assembly of pPro-AG(*lacZ*)
58_RvlacZHA2	gagtttttgttcgggcccaactcatccatgacctgaccatgc	
63_FwP-AG(lacZ)	tggacgagctgtacaagtaacttccctatcagtgatagagattgacatcc	Amplification of 8003 bp (F1), spanning pCRISPR(lacZgRNA +HA1) for Gibson assembly of pPro-AGFP(*lacZ*)
64_RvP-AG(lacZ)	tcctcgcccttgctcaccatcatatcctgatcttccagataactgccg	
65_FwGFP-lacZ	atctggaagatcaggatatgatggtgagcaagggcgag	Amplification of *gfp* sequence (760 bp, F2) for Gibson assembly of pPro-AGFP(*lacZ*)
66_RvGFP-lacZ	ctctatcactgatagggaagttacttgtacagctcgtccatgccg	

67_FwHA1-GFPout	accagtagaaacagacgaagaatcgtggtggcctaactacggct	Amplification of *bla* sequence HA1 (535 bp, F1) for Gibson assembly of pgRNAout(Amp)
68_RvHA1-GFPout	ttgctcaccatttgagatccagttcgatgtaacccac	
69_FwHA2-GFPout	ctgtacaagtaacagcggtaagatccttgagagtttt	Amplification of *bla* sequence HA2 (537 bp, F2) for Gibson assembly of pgRNAout(Amp)
70_RvHA2-GFPout	tatcaaagggaaaactgtccataccgtcctgcaactttatccgcct	
71_FwGFP-GFPout	ggatctcaaatggtgagcaagggcgag	Amplification of *gfp* sequence (742 bp, F3) for Gibson assembly of pgRNAout(Amp)
72_RvGFP-GFPout	atcttaccgctgttacttgtacagctcgtccatgccg	
49_FwlacZseq	agacgcgaattatttttgatggcgttaactcg	Amplification of *lacZ* internal sequence for sequencing editing events. PCR products length varied among insertions (2008 bp for GFP cargo delivered, 1288 bp for gRNA cassette insertion and 1124 bp for escapers)
50_RvlacZseq	gaagggctggtcttcatccacgcg	
63_RvlacZint	gccgctggcgacctgcgtttca	Sequencing of *lacZ* editing events, internal primer that hybridizes within *lacZ* HA2 and downstream *lacZ* gRNA sequences
35_FwAmp(ext)	gagtattcaacatttccgtgtcgc	Sequencing of *bla* editing events, internal primer that hybridizes within *bla* HA1 and downstream AmpgRNA2 sequences
102_FwCRISPR	cagggtagccagcagcatcccttccctatcagtgatagagattgacatcc	Amplification of AmpgRNA2 cassette (203 bp, F1) for Gibson assembly of pETgCRISPR
103_RvCRISPR	tgttccggatctgcatcgcagcttcaaaaaaagcaccgactcg	
104_FwpETg(CRISPR)	gtcggtgctttttttgaagctgcgatgcagatccggaac	Amplification of 6076 bp (F2), spanning pETg for Gibson assembly of pETgCRISPR
105_RvpETg(CRISPR)	ctctatcactgatagggaagggatgctgctggctaccc	
98_FwPro-AG	cagggtagccagcagcatccgtggtggcctaactacggct	Amplification of Pro-AG cassette (1204 bp, F1) for Gibson assembly of pETgPro_AG
99_RvPro-AG	tgttccggatctgcatcgcagtcctgcaactttatccgcct	
100_FwpETg(Pro-AG)	ggcggataaagttgcaggactgcgatgcagatccgga	Amplification of 6076 bp (F2), spanning pETg for Gibson assembly of pETgCRISPR
101_RvpETg(Pro-AG)	agccgtagttaggccaccacggatgctgctggctaccct	
106_FwHA1Supercos	ctctatcactgatagggaagttgagatccagttcgatgtaaccc	Amplification of *bla* sequence HA1 from Super-Cos (SV3B05) (540 bp, F1) for Gibson assembly of pCRISPR (AmpgRNA2+SV3B05HA1)
107_RvHA1Supercos	taaggatgatttctggaatttgaggacgaggtggcc	
108_FwpKDsAmp2	tacatcgaactggatctcaacttccctatcagtgatagagattgacat	Amplification of 7002 bp (F2), spanning pCRISPR(AmpgRNA2) for Gibson assembly of pCRISPR (AmpgRNA2+SV3B05HA1)
109_RvpKDsAmp2	tgacggccacctcgtcctcaaattccagaaatcatccttagcgaaag	
110_Fw HA2Supercos	gagtttttgttcgggcccaagtcctgcaactttatccgcct	Amplification of *bla* sequence HA2 from Super-Cos (SV3B05) (540 bp, F1) for Gibson assembly of pPro-AG Super-Cos
111_Rev HA2Supercos	gtcggtgctttttttgaagccagcggtaagatccttgagagtttt	
Pr_112Fw CosHA1	ggcggataaagttgcaggacttgggcccgaacaaaaact	Amplification of 7502 bp (F2), spanning pCRISPR (AmpgRNA2+SV3B05HA1) for Gibson assembly of pPro-AG Super-Cos
Pr_113Rv CosHA1	tctcaaggatcttaccgctggcttcaaaaaaagcaccgactcg	
88_FwHA1-lacZout	tcaggtttgtgccaataccagtagaaacagacgaagaatcggcgtaatagcga	Amplification of *lacZ* sequence HA1 (560 bp, F1) for Gibson assembly of pgRNAout(*lacZ*)
89_RvHA1-lacZout	tcctcgcccttgctcaccatcatatcctgatcttccagataactgccg	
90_FwGFP-lacZout	atctggaagatcaggatatgatggtgagcaagggcgag	Amplification of *gfp* sequence (760 bp, F2) for Gibson assembly of pgRNAout(*lacZ*)
91_RvGFP-lacZout	aaaatgccgctcatccgccattacttgtacagctcgtccatgcc	
92_FwHA2-lacZout	tggacgagctgtacaagtaatggcggatgagcggca	Amplification of *lacZ* sequence HA2 (559 bp, F3) for Gibson assembly of pgRNAout(*lacZ*)
93_RvHA2-lacZout	gttcaccgttacatatcaaagggaaaactgtccataccctcatccatgacctgacc	
27_Fw pKDSgRNAseq	atcccgtgacaggtcattcagactg	Sequencing of gRNA plasmid contruct from editing events.
28_Rv pKDSgRNAseq	gatttaatctgtatcagg	
